# Genome-wide association studies and meta-analysis uncovers new candidate genes for growth and carcass traits in pigs

**DOI:** 10.1371/journal.pone.0205576

**Published:** 2018-10-11

**Authors:** Iulia Blaj, Jens Tetens, Siegfried Preuß, Jörn Bennewitz, Georg Thaller

**Affiliations:** 1 Institute of Animal Breeding and Husbandry, Kiel University, Kiel, Germany; 2 Functional Breeding Group, Department of Animal Sciences, Göttingen University, Göttingen, Germany; 3 Institute of Animal Husbandry and Breeding, University of Hohenheim, Stuttgart, Germany; INIA, SPAIN

## Abstract

Genome-wide association studies (GWAS) have been widely used in the genetic dissection of complex traits. As more genomic data is being generated within different commercial or resource pig populations, the challenge which arises is how to collectively investigate the data with the purpose to increase sample size and implicitly the statistical power. This study performs an individual population GWAS, a joint population GWAS and a meta-analysis in three pig F_2_ populations. D1 is derived from European type breeds (Piétrain, Large White and Landrace), D2 is obtained from an Asian breed (Meishan) and Piétrain, and D3 stems from a European Wild Boar and Piétrain, which is the common founder breed. The traits investigated are average daily gain, backfat thickness, meat to fat ratio and carcass length. The joint and the meta-analysis did not identify additional genomic clusters besides the ones discovered via the individual population GWAS. However, the benefit was an increased mapping resolution which pinpointed to narrower clusters harboring causative variants. The joint analysis identified a higher number of clusters as compared to the meta-analysis; nevertheless, the significance levels and the number of significant variants in the meta-analysis were generally higher. Both types of analysis had similar outputs suggesting that the two strategies can complement each other and that the meta-analysis approach can be a valuable tool whenever access to raw datasets is limited. Overall, a total of 20 genomic clusters that predominantly overlapped at various extents, were identified on chromosomes 2, 7 and 17, many confirming previously identified quantitative trait loci. Several new candidate genes are being proposed and, among them, a strong candidate gene to be taken into account for subsequent analysis is *BMP2* (bone morphogenetic protein 2).

## Background

In pig breeding, the search for quantitative trait loci (QTLs) and the underlying causative mutations has been in progress for more than two decades. The onset was the landmark publication on genetic mapping of QTL for growth and fatness by [1]. Up to date, according to the latest release of the AnimalQTLdb (Release 35, 29^th^ April, 2018), the Pig QTL database stores 27,465 pig QTLs curated from 620 publications and representing a wide range of economically important phenotypes (PigQTLdb; https://www.animalgenome.org/cgi-bin/QTLdb/SS/summary).

In the beginning of pig QTL experiments, the mapping was carried out using crosses between outbred lines and the statistical test employed was linkage analysis. This approach has proven to be efficient for pinpointing numerous QTLs in pigs [2, 3]. However, those studies had reduced mapping resolution and statistical power due to several factors as: the limited number of individuals and genetic markers, and linkage analysis usage which considers only recent recombination events.

The release of the Illumina PorcineSNP60 Beadchip [4] represents a key advance in overcoming the above-mentioned impediments. The SNP array facilitates the implementation of genome-wide association studies (GWAS) in which the historical recombination events are taken into account when reflecting the associations between markers and phenotypes. To surpass the limitation given by small sample size in some mapping experiments, analyzing several F_2_ resource populations jointly has been proven to be a suitable approach [5]. Stochastic simulations [5] demonstrated that pooling data from multiple F_2_ populations can increase power and mapping precision compared to single population association analysis under the scenario in which the crosses share at least one common founder breed. Thus, one approach to combine data from several populations is a joint population GWAS (JA) which considers a single dataset comprising the merged information from the individual population level. This approach requires access to the complete original dataset (i.e. genotypes and phenotypes). Another strategy for combining information from multiple genetic mapping studies is the meta-analysis (MA) of the GWAS summary statistics. This method can increase the detection power and reduce false-positive findings [6] while also allowing to efficiently account for population substructure and for study specific covariates [7]. The MA is widely used in human genetics, where access to the original datasets is usually limited due to privacy protection policies. In the last years, the meta-analysis has been employed for pig association studies in an effort to maximize the use of available genomic information from commercial or experimental pig populations [8–10].

The current study considers three pig F_2_ resource populations which share a common founder breed [11, 12]. The connecting breed, Piétrain, is an extensively used sire line in pig breeding. Having a constant demand for improving traits related to growth and carcass composition, transferring knowledge from genome-wide association studies results into practice is of utter importance. For this purpose, the classical G-BLUP (genomic best linear unbiased prediction) statistical framework, used for predicting genomic estimated breeding values, was extended to incorporate prior information on QTLs and related biological knowledge via the genomic feature BLUP (GF-BLUP) model [13]. The proposed model is an extension of the linear mixed model used in standard G-BLUP which includes additional genetic effects, previously unraveled by association studies. According to [13], the GF-BLUP can contribute to the prediction accuracy improvement in genomic selection schemes. Considering the above mentioned reasoning, the aim of the study was twofold. Firstly, to conduct a joint design analysis (JA) and a meta-analysis (MA) of the three pig F_2_ resource populations and compare the results yielded by these different approaches. Secondly, to identify candidate genes in genomic regions associated with average daily gain, backfat thickness, meat to fat ratio and carcass length.

## Materials and methods

### Description of resource populations

The three-generation experimental populations comprise a total of 2,380 animals. The designs were established three decades ago by [14] and [15] and for this study, blood samples were available from which DNA was extracted for genotyping purposes. [14] and [15] characterized the populations in detail and will only be described briefly further. The first resource population (D1) considered for this study has 1,785 individuals. It was obtained from five purebred Piétrain (P) boars crossed with one Large White (LW) and six crossbred sows Landrace (L) x Large White (all of them homozygous stress resistant). Large F_2_ families were generated by repeatedly crossing seven F_1_ boars to full-sib F_1_ sows. The second population (D2) is composed of 304 pigs stemming from mating one Meishan (M) boar with eight Piétrain sows. The third population (D3) with 291 individuals had as founders a European Wild Boar (WB) crossed with nine Piétrain sows. Three of the Piétrain sows were common among the latter two families. For both D2 and D3, the F_2_ individuals were the result of two or three F_1_ boars mated with F_1_ sows. Generally, each sow had two litters from different boars. The Piétrain founder females were homozygous stress susceptible and the Meishan and wild boar males were homozygous stress resistant.

### Phenotypic trait data

For the current study the following growth and carcass composition traits were considered: average daily gain (ADG), back fat thickness (BFT), meat to fat ratio (MFR) and carcass length (CRCL). The ADG [g] is the daily weight gain in the fattening period, the BFT [mm] is calculated as the average of three measurements: shoulder fat depth, back fat depth and loin fat depth, the MFR [ratio] is the fat area in relation to the meat area at 13^th^/14^th^ rib and CRCL [cm] is measured from the first cervical vertebrae to the pubis symphysis. The methods of measurement and the calculations employed for D1, D2 and D3 were in conformity with the performance testing directive of the Central Association for German Pig Production [16, 17]. Table 1 contains a brief description of the contributing F_2_ designs and the summary of the phenotypic data indicating mean and standard deviation (SD) for each trait. The animals were slaughtered at 211.01 ± 22.3 days, 211.73 ± 6.92 days and 210.63 ± 3.22 for D1, D2 and D3, respectively.

**Table 1:** Description of the experimental F_2_ populations and investigated traits.

	D1: Px(LxLW)/LW^a^ Males F_0_ = 5 Females F_0_ = 7		D2: MxP^b^ Males F_0_ = 1 Females F_0_ = 8		D3: WBxP^c^ Males F_0_ = 1 Females F_0_ = 9	
	Mean (SD)	N^d^	Mean (SD)	N^d^	Mean (SD)	N^d^
ADG[g]^e^	675.9 (92.74) 311–1039	1769	590.1 (130.03) 174.0–951.0	304	527.4 (109.17) 125.0–790.0	291
BFT[mm]^e^	27.49 (3.84) 16–42.3	1766	27.89 (6.73) 8.70–46.00	304	22.8 (4.97) 10.3–40.0	291
MFR[ratio]^e^	0.38 (0.11) 0.14–0.85	1765	0.7248 (0.22) 0.28–1.39	304	0.516 (0.08) 0.19–1.07	289
CRCL[cm]^e^	100 (2.93) 91–111	1765	91.33 (6.12) 63.50–106.00	304	79.85 (5.20) 62.50–94.00	291

^a^Piétrain x (Landrace x Large White)/Large White;

^b^Meishan x Piétrain;

^c^Wild Boar x Piétrain;

^d^Number of individuals with phenotypic data;

^e^Average daily gain, backfat thickness, meat to fat ratio, carcass length.

### Genotyping and quality control

The F_2_ individuals were genotyped with Illumina PorcineSNP60 BeadChip (61,565 SNPs). SNP chromosomal positions were based on the current pig genome assembly (Sus Scrofa build 11.1 provided by Swine Genome Sequencing Consortium on NCBI). Genotypes were filtered with respect to the following quality control (QC) criteria: i) removing SNPs with a minor allele frequency less than 5% and ii) excluding individuals and SNPs with call rates lower than 90%. The process of quality control was carried out using Plink [18]. The autosomal chromosomes were further considered. The final set consisted of 44,457 SNPs in D1 design, 40,738 SNPs in D2 design, 37,145 in D3 design and 31,299 SNPs in the joint design (D1D2D3). The latter was obtained by merging common SNPs from D1, D2 and D3 after the QC step. In addition, the *RYR1*:g.1843C>T [19] mutation status was available for the individuals in D2 and D3.

### Persistence of linkage disequilibrium phase

The extent of linkage disequilibrium (LD) in the D1, D2, D3 and D1D2D3 populations was characterized in detail by [20]. Of further interest for the joint analysis was to examine how consistent is the LD phase in the designs. The statistical parameter chosen for the LD measurement was *r*^*2*^ [21], which is the correlation coefficient between SNP pairs. A total of 31,299 common SNP across the individual populations and the joint data set was used to compute the *r*^*2*^ values. Using Plink, *r*^*2*^ was obtained for all SNP pairs located less than 5 Mb apart. The average *r*^*2*^ values of the SNP pairs in classes of inter-marker distances of 100 Kb starting with interval [0–100] Kb up to [4900–5000] Kb was calculated and finally used to compute correlation of phase between two populations according to the formula [22]:

$$R_{D_{k},D_{k\prime }}=\frac{\Sigma_{(i,j)\epsilon p}(r_{ij(D_{k})}-{\overset{-}{r}}_{\left(D_{k}\right)})(r_{ij(D_{k}\prime )}-{\overset{-}{r}}_{\left(D_{k\prime }\right)})}{S_{{(D}_{k})}S_{{(D}_{k\prime })}}$$

where $R_{D_{k},D_{k\prime }}$ is the correlation of phase between $r_{ij(D_{k})}$ in population *D*_*k*_ and $r_{ij(D_{k}\prime )}$ in population *D*_k′_, $S_{{(D}_{k})}$ and $S_{{(D}_{k\prime })}$ are the standard deviation of $r_{ij(D_{k})}$ and $r_{ij(D_{k}\prime )}$, respectively, and the average *r*_*ij*_ across all SNP *i* and *j* within interval *p* for *D*_*k*_ and *D*_*k*′_, accordingly, is denoted with ${\overset{-}{r}}_{\left(D_{k}\right)}$ and ${\overset{-}{r}}_{\left(D_{k\prime }\right)}$. The $R_{D_{k},D_{k\prime }}$ estimate was evaluated for the six population pairs: D1-D2, D1-D3, D1-D1D2D3, D2-D3, D2-D1D2D3 and D3-D1D2D3.

### Estimating genetic variance and genetic correlations

A general linear model was used to pre-adjust the animal phenotypes with the following fixed effects: sex, stable and slaughter month class (i.e. 15 classes for D1, 6 classes for D2 and 8 classes for D3). The phenotype pre-adjustment analysis was carried out with R [23]. For D2 and D3 the *RYR1* status was also incorporated as fixed effect for ADG, BFT and MFR traits. Weight at slaughter was included as a covariate for all traits except for ADG. The residual values of the phenotypes were subsequently used. The Genome-wide Complex Trait Analysis tool (GCTA) [24] was utilized for estimating the variance components and the genetic correlations. The genomic relationship matrix (GRM) was calculated between all pairs of individuals using all the autosomal SNPs. Applying a univariate analysis, the variance of the traits was partitioned using restricted maximum likelihood (REML) into an additive genetic and a residual component. Following the classical definition of narrow-sense heritability, the SNP-based heritability was obtained via $h_{SNP}^{2}=\sigma_{SNP}^{2}/\sigma_{P}^{2}$, representing the proportion of phenotypic variance ($\sigma_{P}^{2}$) explained by the additive effects of the common SNPs on the chip array and/or by the unknown causal variants correlated with the SNPs. A bivariate GREML analysis led to the assessment of the genetic correlations between traits.

### GWAS and meta-analysis

Single marker association tests were performed using the GCTA tool for the individual populations (i.e. D1, D2 and D3). A mixed linear model analysis including the candidate SNP (MLMi) was set up as follows: y* = *xb* + *u* + *e*, where y* is the phenotype corrected for systematic environmental effects and genetic effect (i.e. the *RYR1* status for D2 and D3), *x* is the genotypes of the marker, *b* is the additive effect size (fixed effect) of the candidate SNP to be tested for association, *u* is the random polygenic effect given by the construction of the genomic relationship matrix (GRM) and *e* is the residual effect.

For the joint design (D1D2D3), the same MLMi was used. The y*, in this case, consisted of pooled pre-corrected phenotypes from D1, D2 and D3. From the unique genotype file, constructed based on the merged common SNPs among the three populations, the GRM was assembled and then the marker genotypes tested for association. Given the fact that D1D2D3 contains strong familial relatedness (due to full-sib families) and weak population stratification, observed in a multidimensional scaling analysis by [20], the mixed linear model analysis should be efficient in capturing sample structure via the GRM as the random effect included in the model [25]. Nevertheless, a fixed effect with three classes representing the three individual designs was added to the model to account for the diverse genetic backgrounds.

A meta-analysis aims to statistically combine the information from multiple independent studies and therefore to increase the power and reduce the false-positive results [6]. From the several approaches to conduct a meta-analysis, the fixed effects meta-analysis is the most powerful method and within this group, the inverse variance based strategy is predominant [6]. This strategy was employed for synthesizing the association studies summary statistics for the common variants of D1, D2 and D3 populations. Specifically, using the METAL software [7], each study was weighted according to the inverse of its squared standard error resulting in newly derived effect sizes and standard errors estimates further used for calculating an overall Z score and finally the overall *p* values.

Manhattan plots for D1, D2, D3 and D1D2D3 GWAS as well as for the MA results were created via *qqman* R package [26]. By using Bonferroni correction, the genome-wide significance line was set to *p*_*genome*-*wide*_ ≤ 0.05. Because Bonferroni correction acts in a stringent manner, an additional nominal significant level was used for which the threshold was set up to *p* ≤ 5*x*10^−5^. The R package *qvalue* [27] facilitated the calculation of the false discovery rate (FDR) *q* value for each association test. The FDR *q* value of the significant SNP with the largest *p* value provided an assessment of the proportion of false positives among the significant SNPs.

Clusters incorporating strong evidence for trait-associated chromosomal regions were defined based on the LD structure and the significant SNPs similar to [20]. A cluster enclosed a minimum of two genome-wide significant SNPs with a maximum distance of 2 Mb between them. From the center point of the initially defined cluster, the upper and the lower boundaries were assigned to the last nominally significant variant situated at a maximum of 1 Mb in both directions.

The jvenn tool [28] was used to draw Venn diagrams for all the SNPs surpassing the nominal significance threshold to show all possible relations for each trait and between the five different sets (D1, D2, D3, D1D2D3 and MA).

### Exploratory analysis of clusters

The clusters identified were explored using BioMart tool [29], the Ensembl Genes 91 database [30] and Gene Ontology [31, 32]. The interrogations were carried out using the latest genome reference, the Sscrofa 11.1 assembly (GCA_000003025.6) and the latest gene annotation (Genebuild released on July 2017).

## Results

### Genotypic data and individuals qualified for the analysis

All genotyped animals passed the quality control procedure, i.e. 1,785 in D1, 304 in D2 and 291 in D3. The final autosomal number of SNPs was 44,457, 40,738 and 37,145 for D1, D2 and D3, respectively. Based on the reference genome assembly (Sscrofa 11.1) the average physical spacing between adjacent markers was 50,872 bp in D1, 55,495 bp in D2 and 60,821 bp for D3. For both the joint design and the meta-analysis, 31,299 SNPs were used with an average physical distance among adjacent markers of 72,161 bp.

### LD and persistence of phase

**Fig 1:**
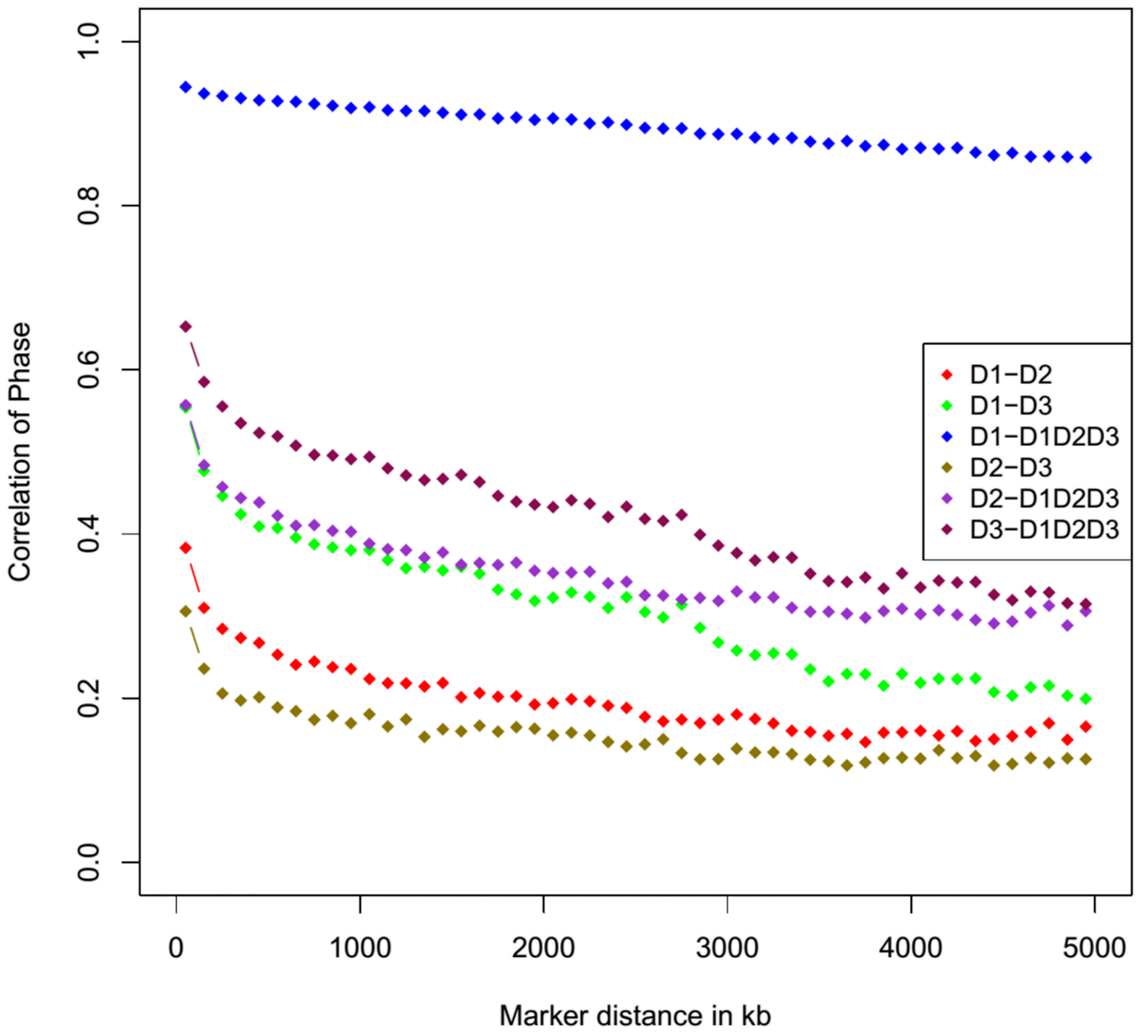
Correlation of phase between D1-D2, D1-D3, D1-D1D2D3, D2-D3, D2-D1D2D3 and D3-D1D2D3 populations for SNP pairs at varying distances.

Average genome-wide *r*^2^ value for adjacent markers was 0.40, 0.44, 0.45 and 0.38 for D1, D2, D3 and D1D2D3, respectively. The correlation of phase $R_{D_{1},{D_{1}D}_{2}D_{3}}$ exhibited high concordance at all marker intervals, starting with 0.95 for the interval [0:100] Kb and maintained values above 0.86 until the maximum interval length considered for the analysis (Fig 1). The remaining five design pairs had visibly lower correlations levels ranging from 0.65 for D3-D1D2D3 pair to 0.31 for D2-D3 when considering the first interval [0:100] Kb. The D2 design was the least correlated with the joint design. Among the individual design pairs (i.e. D1-D2, D1-D3 and D2-D3), the highest persistence of phase was observed for D1-D3 and the lowest for D2-D3 pair.

### Heritabilities and genetic correlations

The SNP-based heritabilities calculated were moderate to high, ranging from 0.31 for CRCL in D3 to 0.74 for CRCL in D2 (Table 2). The $h_{SNP}^{2}$ estimates for ADG were highest in D3 and for BFT and CRCL in D2. The values for MFR were constant in all three designs. The traits BFT and MFR were strongly positive correlated with a coefficient *r*_*G*_ = 0.77 in D1, *r*_*G*_ = 0.53 in D2 and *r*_*G*_ = 0.83 in D3, while for BFT and CRCL a mild to strong negative correlation were observed (i.e. *r*_*G*_ = -0.29 in D1, to *r*_*G*_ = -0.78 in D2 and *r*_*G*_ = -0.46 in D3).

**Table 2:** SNP-based heritabilities ($h_{SNP}^{2}$) on the diagonal and genetic correlations on the lower triangle (with standard errors).

Design	Trait	ADG	BFT	MFR	CRCL
**D1**	**ADG**	0.36 (0.04)			
	**BFT**	-0.10 (0.09)	0.45 (0.04)		
	**MFR**	-0.12 (0.09)	0.77 (0.04)	0.52 (0.03)	
	**CRCL**	-0.07 (0.08)	-0.29 (0.07)	-0.04 (0.07)	0.60 (0.03)
**D2**	**ADG**	0.33 (0.10)			
	**BFT**	-0.35 (0.17)	0.73 (0.07)		
	**MFR**	-0.41 (0.19)	0.53 (0.12)	0.47 (0.09)	
	**CRCL**	0.42 (0.17)	-0.78 (0.06)	-0.20 (0.15)	0.74 (0.06)
**D3**	**ADG**	0.57 (0.09)			
	**BFT**	-0.36 (0.17)	0.42 (0.09)		
	**MFR**	-0.31 (0.16)	0.83 (0.08)	0.47 (0.09)	
	**CRCL**	0.31 (0.20)	-0.46 (0.21)	-0.26 (0.21)	0.31 (0.10)

### Variants identified in D1, D2, D3, D1D2D3 GWAS and MA

**Fig 2:**
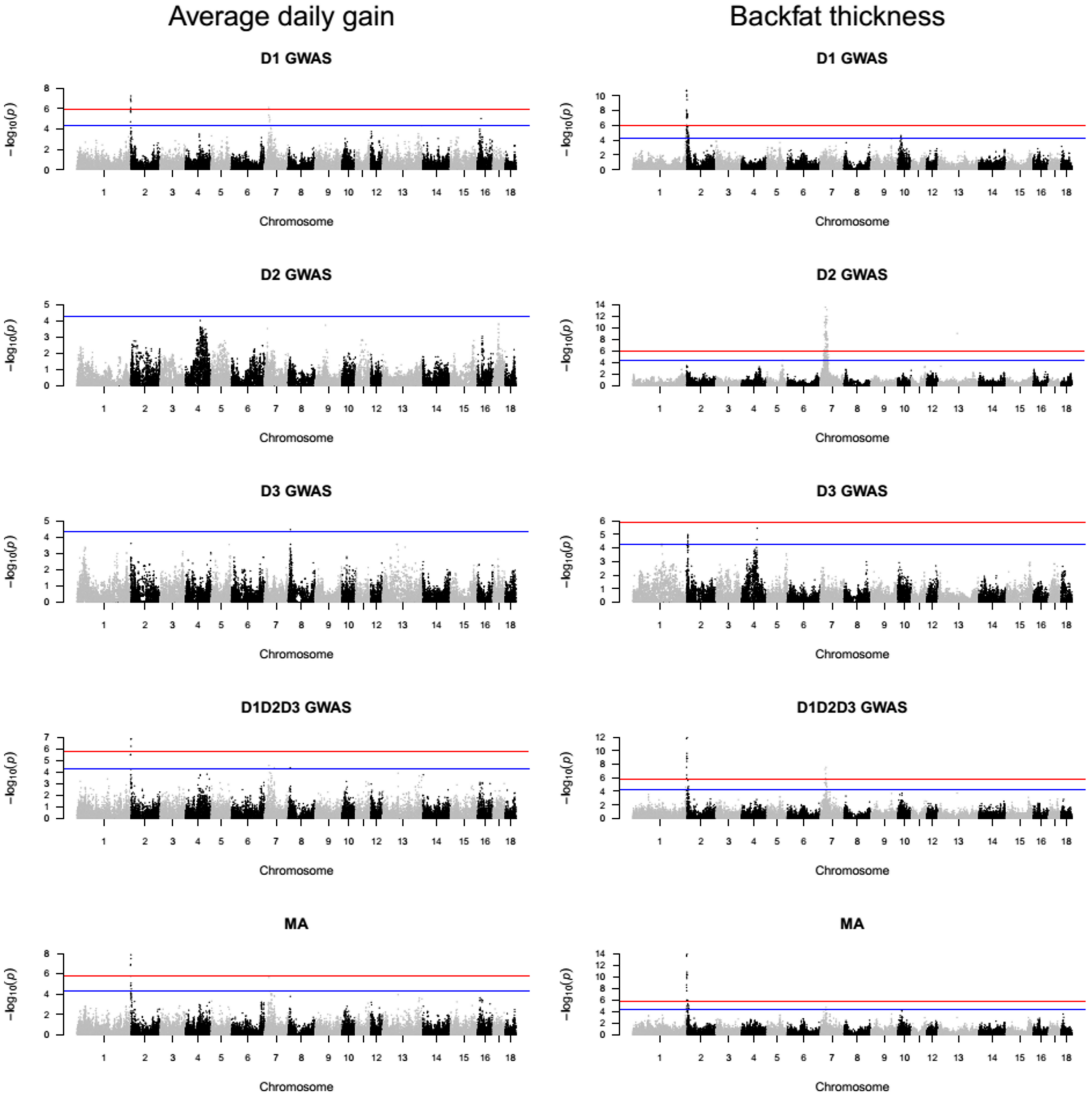
Manhattan plots of the −log_10_ *p* values for association of SNPs with ADG and BFT in D1, D2, D3 and D1D2D3 GWAS and MA. The top horizontal line indicates the genome-wide significance level *p*_*genome-wide*_ ≤ 0.05 and the bottom line indicates the nominal level of significance *p*_*nominal*_ ≤ 5*x*10^−5^.

**Fig 3:**
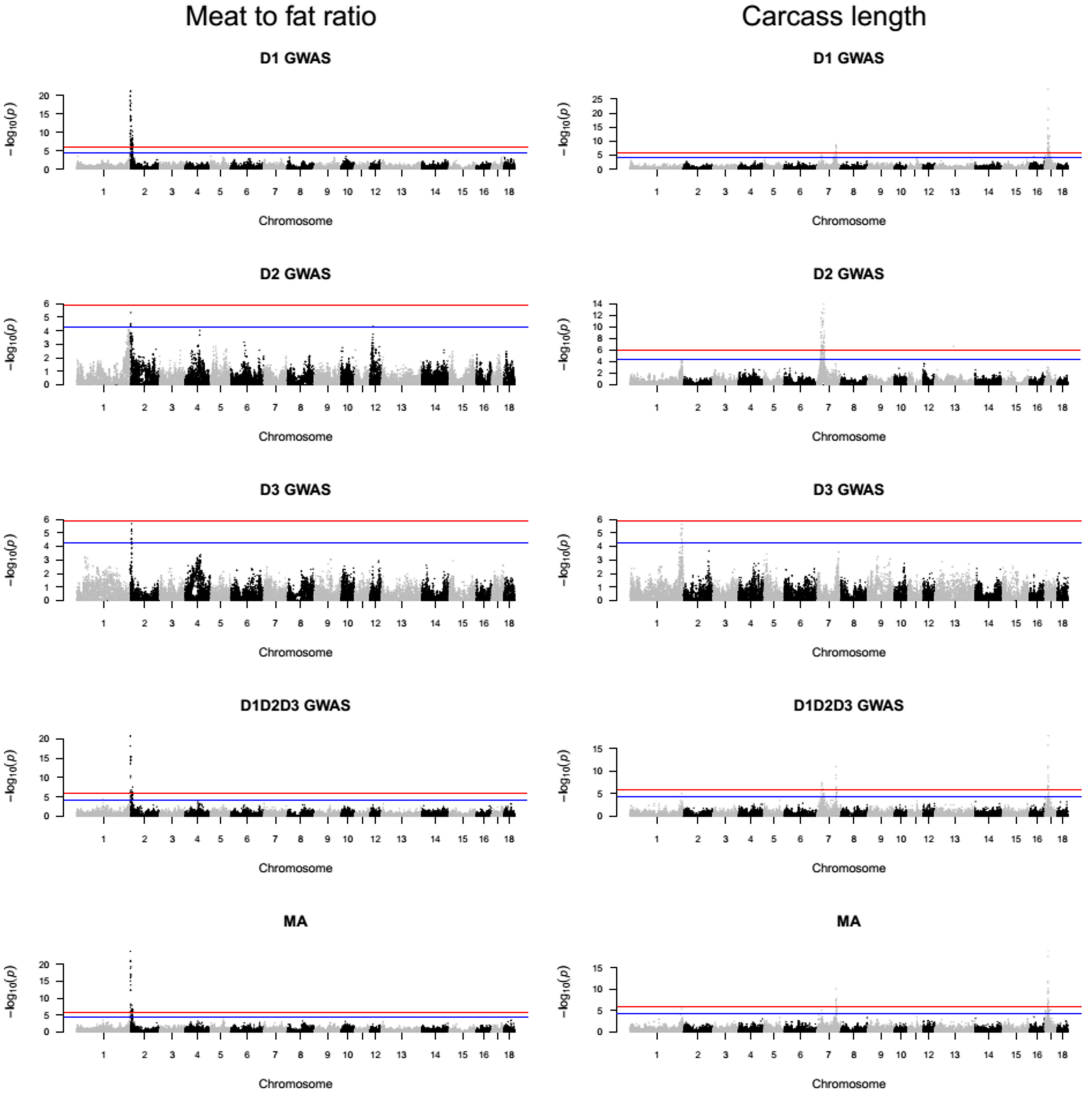
Manhattan plots of the −log_10_ *p* values for association of SNPs with MFR and CRCL in D1, D2, D3 and D1D2D3 GWAS and MA. The top horizontal line indicates the genome-wide significance level *p*_*genome-wide*_ ≤ 0.05 and the bottom line indicates the nominal level of significance *p*_*nominal*_ ≤ 5*x*10^−5^.

A GWAS was conducted on the D1, D2, D3 and D1D2D3 designs for four phenotypic traits (i.e. ADG, BFT, MFR and CRCL). The summary statistic from the D1, D2 and D3 association analysis was further used for the MA step. The global view of *p* values for all SNP markers of each trait was visualized via a Manhattan plot (Figs 2 and 3). Cumulated over the five analyses (i.e. D1 GWAS, D2 GWAS, D3 GWAS, D1D2D3 GWAS and MA), for ADG a total of 36 SNPs surpassed the nominal significance level from which 14 SNPs were above the genome-wide significance level. These variants were situated on the *Sus Scrofa* chromosome (SSC) 2, 7, 8, 13 and 16. For BFT, 299 (with 148 above genome-wide significance level) variants were identified on the following SSC: 1, 2, 4, 5, 7, 10 and 13 and for MFR, 223 (with 137 above genome-wide significance level) SNPs located on SSC 1, 2 and 12. Lastly, for CRCL, 368 (with 193 surpassing genome-wide significance level) SNPs on SSC 1, 7, 13, 16 and 17 were discovered via the GWAS and the MA. The top significant variants in all the five analyses conducted are presented in Table 3 together with their—log_10_ *p* value and the associated *q* value. The genomic regions with genome-wide significant and nominal significant SNPs (S1 Table) were assigned to a total of 20 clusters (Table 4).

**Table 3:** List of the highest significant SNP from all five analyses and for all four traits.

Trait	GWAS or MA	Top SNP	SSC^a^	Location bp	-log10 (*p* value)	*q* value	Total SNPs^b^	Other SSC^c^
**ADG**	D1	ALGA0123907	2	2,556,939	7.26	0.00206	16 (6)	7, 13, 16
	D2	H3GA0013062	4	74,916,258	4.03	0.47904	-	-
	D3	H3GA0024295	8	11,805,802	4.47	0.97386	1	-
	D1D2D3	ALGA0123907	2	2,556,939	6.85	0.00224	8 (3)	7, 8
	MA	ALGA0123907	2	2,556,939	8.16	0.00020	11 (5)	7
**BFT**	D1	ASGA0085597	2	1,083,343	10.71	3.29e-07	48 (22)	1, 10
	D2	INRA0024524	7	26,069,284	13.56	1.12e-09	179 (93)	5,13
	D3	INRA0015172	4	79,915,989	5.47	0.09920	10	1, 2
	D1D2D3	ASGA0085597	2	1,083,343	12.14	1.68e-08	34(16)	1, 7
	MA	ASGA0008415	2	3,895,569	14.01	2.74e-10	28 (16)	1, 7
**MFR**	D1	MARC0044928	2	2,494,326	21.28	1.93e-17	103 (76)	1
	D2	ASGA0089068	2	3,237,229	5.35	0.16883	7	12
	D3	ALGA0011643	2	7,557,050	5.70	0.04177	15	-
	D1D2D3	ASGA0085597	2	1,083,343	20.84	2.89e-17	43 (22)	1
	MA	ASGA0085597	2	1,083,343	24.04	1.63e-20	55 (39)	1
**CRCL**	D1	MARC0070553	17	15,827,832	28.68	9.25e-25	98 (47)	7,16
	D2	DIAS0000554	7	34,166,932	14.04	3.68e-10	146 (93)	13
	D3	H3GA0004878	1	265,179,997	5.65	0.04440	11	-
	D1D2D3	ALGA0093478	17	16,919,581	17.88	4.08e-14	56 (29)	1, 7
	MA	ALGA0093478	17	16,919,581	19.08	2.59e-15	57 (26)	1, 7

^a^*Sus Scrofa* chromosome;

^b^Total number of SNPs significant at a nominal level (total number SNPs significant at a genome-wide level);

^c^Other chromosomes on which associations surpassing the nominal significance level were detected.

**Table 4:** Number of genomic clusters, localization and number of significant SNPs.

Trait	Analysis GWAS/MA	Cluster number	SSC^a^	Cluster boundaries (bp)	Length in Mb	Number of significant SNPs^b^
**ADG**	D1	1	2	631,324–2,586,096	1.94	7 (5)
	D1D2D3	2	2	2,556,939–2,586,096	0.03	3 (3)
	MA	3	2	141,798–2,586,096	2.44	9 (5)
**BFT**	D1	4	2	236,179–5,189,397	4.95	31 (23)
	D2	5	7	19,567,933–37,145,252	17.58	165 (92)
	D1D2D3	6	2	141,798–3,895,569	3.75	13 (13)
		7	7	26,522,116–28,252,780	1.73	5 (2)
	MA	6	2	141,798–3,895,569	3.75	17 (15)
**MFR**	D1	8	2	70,140–13,307,467	13.24	98 (76)
	D1D2D3	6	2	141,798–3,895,569	3.75	18 (16)
		9	2	7,536,991–8,647,689	1.11	6 (2)
		10	2	12,168,412–13,294,789	1.13	11 (3)
	MA	11	2	141,798–10,254,380	10.11	37 (30)
		12	2	12,275,048–13,294,789	1.02	9 (8)
**CRCL**	D1	13	7	97,195,350–99,887,568	2.69	15 (10)
		14	17	12,361,530–19,474,175	7.11	42 (27)
		15	17	21,832,087–23,773,788	1.94	11 (10)
	D2	16	7	19,567,933–36,795,710	17.23	135 (92)
	D1D2D3	17	7	23,659,424–26,522,116	2.86	8 (5)
		18	7	97,147,161–99,424,987	2.28	9 (7)
		19	17	13,692,477–19,474,175	5.78	21 (16)
	MA	20	7	97,147,161–99,491,117	2.34	10 (7)
		19	17	13,692,477–19,474,175	5.78	27 (17)

^a^*Sus Scrofa* chromosome;

^b^Total number of SNPs significant at a nominal level (total number SNPs significant at a genome-wide level).

**Fig 4:**
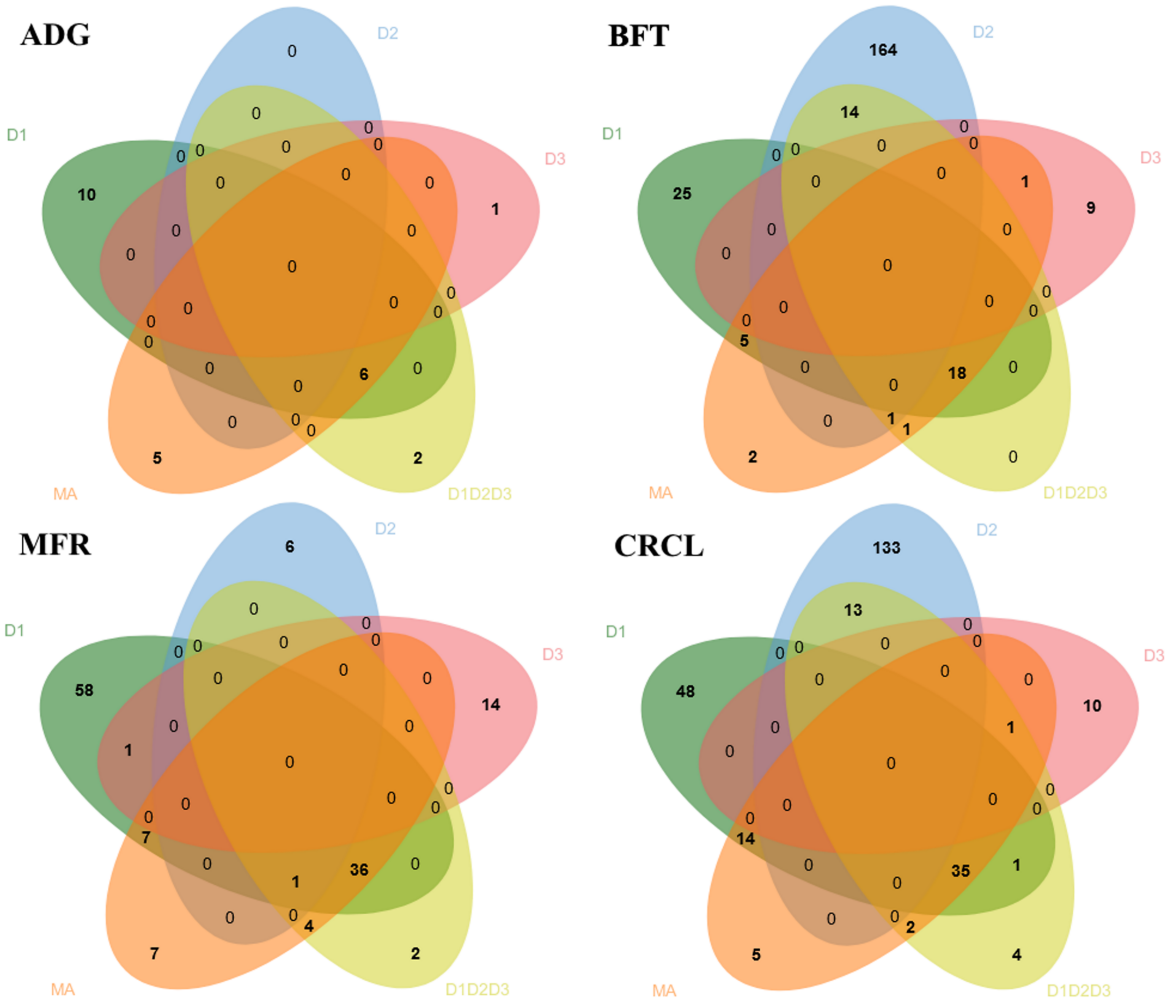
Venn diagram displaying common variants identified for the four traits via the five analyses (i.e. D1, D2, D3, D1D2D3 GWAS and MA). By using Pearson’s product-moment correlation, the accordance of the *p* values was estimated by selecting the common set of markers between the output from the association studies conducted for D1, D2, D3, D1D2D3 and the meta-analysis results (S2 Table). The correlation value between JA and MA *p* values was 0.83 for ADG, 0.81 for BFT, 0.74 for MFR and CRCL (all significant with *p* < 0.05). The D1 *p* values were highest correlated with the JA and the MA as compared to the D2 and D3 designs’ *p* values.

The concordance of the nominally significant variants identified was assessed via Venn diagrams (Fig 4). The meta-analysis revealed more unique SNPs associated to the traits as compared to the joint analysis. Specifically, five SNPs for ADG and CRCL, two SNP for BFT and seven SNPs for MFR were identified exclusively by the meta-analysis. One variant for BFT, four variants for MFR and two variants for CRCL were common elements found only by the joint and meta-analysis. While many variants from the individual population association studies overlapped with variants from JA and MA, the individual populations showed a higher number of unshared variants for the all traits.

## Discussion

Linkage disequilibrium between markers and quantitative trait loci is fundamental for conducting a successful genome-wide association study. In order to disentangle variants associated with complex traits, the LD pattern in the populations under investigation must be evaluated. This particular analysis was carried out by [20] for the D1, D2 and D3 populations included in this study. The main findings were that there is a faster LD decay in the European type breeds cross (D1) as compared to the Asian/Wild Boar and European breeds cross (D2 and D3), while the fastest breakdown of LD is observed by pooling the data. The latter finding is supportive of the fact that the joint design (D1D2D3) could have a positive impact on the mapping resolution. Also in accordance to this study were the results by [33] and [5] obtained via stochastic simulations of populations with a similar phylogeny as D1, D2 and D3.

The linkage phases between SNPs and the causative mutations that underlie the detected QTL are not always identical across populations. The D1, D2 and D3 F_2_ populations are established from genetically divergent breeds of Asian (Meishan) and European origin (Landrace, Large White, Piétrain—the common founder breed) as well as the European Wild Boar ancestor. Introgression of Asian pigs into the European stocks has been well documented during the 18^th^ and 19^th^ century, fact which led to the existence of Asian haplotypes within the European commercial breeds for traits such as backfat and litter size [34]. Therefore, the premise of having shared QTLs for some of the investigated traits among the populations is supported. Considering this, an additional method to assess the feasibility of conducting a joint analysis depends on how consistent is the LD phase in the individual designs as compared to the LD phase in the joint dataset. Across all population pairs (i.e. D1-D2, D1-D3, D1-D1D2D3, D2-D3, D2-D1D2D3 and D3-D1D2D3), the phase correlation decreased with increasing marker distance (Fig 1). Or alternatively stated, the shorter the chromosomal segment, the greater the chance of the LD phase to be similar. As longer distances are considered, there is a higher chance for recombination events to disrupt the LD which was present in the ancestral population as new LD is formed within the derived subpopulations [21]. The phase agreement between D1-D1D2D3 had correlation values ranging from 0.86 to 0.96 due to the fact that 75% of the joint design is composed of D1 individuals and their overall allele frequencies prevail when pooling the three designs. The second most correlated pair, D3-D1D2D3, contains only individuals of European ancestry derived from breeds Piétrain, Large White, Landrace and the European Wild Boar. The least correlated individual design with the joint design was the D2 population which stems from Meishan (Asian) and Piétrain. Nevertheless, for the classes of inter-marker distances less than 500 Kb the correlation of phase was higher than 0.44 suggesting that for shorter chromosome lengths there are LD similarities among these populations. When considering the individual population pairs, the different genetic background was responsible for the low levels of phase agreement in D1-D2 and D3-D2.

Meta-analysis of genome-wide association studies results can increase the power to detect association signals by increasing sample size. The use of this approach grew substantially in the genomics field in the last decade as the scientific community recognized the value of collaborating to combine genetic resources [6, 8–10]. The output of the inverse variance based meta-analysis strategy is dependent on the standard errors of each SNP in each of the study because the weight assigned to each variant is being calculated as the inverse of the squared standard error. Therefore, studies with higher standard errors will have a smaller weight in the meta-analysis. Considering the individual populations GWAS summary statistics, the D2 population has overall higher standard errors as a result of higher phenotypic variance (Table 1). This implies that this study has a smaller weight in the MA, however, this aspect is compensated to a certain degree by the high effect sizes of the associated variants particularly when considering the traits BFT and CRCL, where highly significant associations were detected. Hence, factors influencing the meta-analysis output are the standard error and the effect size which greatly depend on the genetic architecture of the trait under investigation.

One of the main objectives of this study was to compare the meta-analysis with the joint analysis, in which the common individual level genotypes are combined into a single dataset before the association study. Therefore, the agreement among the *p* values was assessed. The correlation value between JA and MA *p* values was higher than 0.7 (*p* < 0.05) for all traits suggesting that the significance levels were similar in the two analysis. Moreover, the *p* values from the individual population association studies were also compared with the results from the joint and meta-analysis. It was observed that the D1 *p* values were the most correlated to the JA (strong persistence of LD phase) and the MA *p* values, while D2 and D3 showed low levels of correlation. A limitation when assessing the agreement of the individual designs with the JA and MA is that the correlation only considers common variants between all five analyses. Some variants which could be highly associated in the individual populations might be disregarded due to being monomorphic in the others; however the correlations value gives a valuable overview at a genome-wide scale of the majority of the SNPs (i.e. 31,299).

From the joint analysis summary statistic a total of eight clusters were assigned and from the meta-analysis output, six (Table 4). Clusters 7 and 17 were identified only by the JA. Many of the significant regions overlapped (S1 Fig) or were identified via both analysis (i.e. Cluster 6 and 19). Except for CRCL, the MA had higher significance levels of the SNPs surpassing the nominal threshold and the clusters were supported by a higher number of variants. The size of the clusters identified was generally smaller for the joint and meta-analysis as compared to the individual population clusters. This suggests that both these approaches have a positive impact on the mapping resolution, pinpointing to narrower locations of causative variants.

### Genetic variance and correlations

In the current study, growth (ADG) and carcass traits (related to fatness: BFT and to anatomy: MFR and CRCL) were investigated. The previous reported heritabilities range from 0.03–0.49 for ADG, 0.12–0.74 for BFT and 0.55–0.60 for CRCL [35]. There are limited resources for the MFR trait represented by only seven QTL listed up to date in the PigQTLdb. The SNP-based heritabilities (Table 2) were overall moderate to high and mostly in accordance to the literature, except for CRCL in D2 ($h_{SNP}^{2}=0.74$), ADG in D3 ($h_{SNP}^{2}=0.57$) and CRCL in D3 ($h_{SNP}^{2}=0.31$). The genetic correlations were high between BFT and MFR (0.77 in D1, 0.53 in D2 and 0.83 in D3) as both traits have a genetic architecture composed of genes involved in the fat metabolism.

### Cluster identification and candidate genes

A total number of 20 genomic clusters were identified (Table 4 and S1 Table). They were located on SSC2, SSC7 and SSC17, and the majority of them overlapped to different extents, except for Cluster 15. Three clusters were found for ADG, four for BFT, six for MFR (one overlapping with a BFT cluster) and eight for CRCL in the D1 GWAS, D2 GWAS, D3 GWAS, JA and MA. The length of the segments varied substantially from 0.03 Mb (Cluster 2 supported by 3 significant SNPs) to 17.58 (Cluster 5 supported by 165 significant SNPs). The long size of the clusters can be attributed to the fact that there are high levels of LD between SNPs which leads to positive signals of association over large genomic regions. Furthermore, the longest clusters (i.e. 5 and 16) were pinpointed for the D2. This appears as a consequence of the small population size which implies fewer meiotic events considered and therefore a lower recombination probability between variants and causative mutations. The results of the joint and meta-analysis did not reveal any new non-overlapping clusters with the ones identified via the single population association study (S1 Fig). Nevertheless, the clusters for the joint and meta-analysis span over shorter genomic regions, pinpointing to more precise locations to identify candidate genes.

The traits in this study are influenced by several genes expressed during the prenatal and postnatal development. Carcass length is a trait mostly determined prenatally and proportional to the length of the spine, as well as the individual length of the vertebrae [36]. Average daily gain, backfat thickness and meat to fat ratio are primarily influenced by tissue growth which can be obtained through cell hyperplasia (e.g. cell proliferation) or hypertrophy (i.e. growth in size) [37].

Several known genes that have their functions previously reported were associated to the traits. One of the most prominent genes, the *IGF2* was not assembled in the Sscrofa 10.2 genome version, but now has been positioned on the Sscrofa 11.1 reference genome. *IGF2* has been described to have an effect on muscle mass and fat deposition [38]. The region where *IGF2* resides is included on six of the clusters identified for ADG, BFT and MFR (1, 3, 4, 6, 8 and 11) which are partially overlapping (S1 Fig). The highest significant SNP in the vicinity of *IGF2* (2:1,469,104–1,496,346) was identified for MFR via meta-analysis: ASGA0085597 (with—log10 (*p* value) = 24.04 and *q* value = 1.63e-20). Moreover, the partially overlapping clusters located on SSC2 (Table 4) harbor genes with growth factor activity (GO: 0008083): *FGF3* (2:3,489,208–3,499,055), *FGF4* (2:3,525,321–3,528,873), *FGF19* (2:3,583,027–3,587,825) and *VEGFB* (2:7,871,217–7,876,372) as well as genes responsible for the maintenance of gastrointestinal epithelium (GO: 0030277): *MUC6* (2:629,678–658,020), *MUC2* (2:711,596–719,372), *MUC5AC* (2:757,933–789,977) and *MUC5B* (2:810,336–841,538). Other genes underlined for ADG, BFT and MFR are *HRAS* (2:299,660–302,501, positive regulation on cell proliferation GO: 0008284) and *DHCR7* (2:2,357,714–2,394,851, lipid metabolic process GO: 0006629). Cluster 10 and 12 found via the joint and meta-analysis narrowed down a region specific for MFR which contains associations for several olfactory receptors which reside in the genomic region SSC2: 12–14 Mb, gene family which is known to have significant expansion throughout time within the pig genome [39].

On SSC7 from 19 to 38 Mb, clusters 5 and 7 showed associations with BFT for the D2 population and in the joint design. Several other studies pinpointed QTLs related to fat traits in the same region [40, 41]. The gene *PPARD* (7:31,222,487–31,297,939) was found as a good gene candidate for fat deposition traits [42]. One of the significant SNPs on cluster 5 (H3GA0020846 with—log10 (*p* value) = 6.11 and *q* value = 3.5e-4) is located in one intron of the gene. Furthermore, the critical region on SSC7 harboring the two clusters for BFT overlaps with the clusters 16 and 17 which were assigned to CRCL in D2 and in the joint analysis, respectively. The common significant variants associated within these clusters for BFT and CRCL also demonstrate discordant direction of effects. This suggests the great interplay between BFT and CRCL associated variants leading to pleiotropic consequences on the phenotypes. It is then reasonable to believe that in prenatal developmental stages the horizontal growth of the animal is already mainly determined by the number of vertebrae and their length while the same or other genes contribute conversely in postnatal existence of the individual ensuring the vertical growth of the backfat thickness. For that reason, the following genes which are located in a highly associated region for both BFT and CRCL (SSC7: 24–26 Mb) were suggested: *BMP5* (7:25,344,729–25,469,988) part of the transforming growth factor-beta (TGF-beta) signaling pathway, *HMGCLL1* (7:25,596,716–25,783,612, ketone body biosynthetic process GO: 0046951), *GFRAL* (7:25,751,185–25,808,637), a receptor required for *GDF15* (Growth differentiation factor 15) mediated reductions in food intake and body weight in mice with obesity [43] and *HCRTR2* (7:25,830,273–25,930,645, feeding behavior GO: 0007631).

Cluster 13, 18 and 20, which mostly overlapped, contained significant associations for carcass length for D1 design, for the joint analysis and the meta-analysis. This genomic region contains genes which have been already associated with carcass length: *VRTN* (7:97,614,707–97,624,273) [44], *LTBP2* (7:97,746,110–97,852,479) [45] and *TGFB3* (7:99,133,511–99,162,221) [46]. The three genes influence the development of vertebrae and ribs in mammalian embryos and thus having a direct influence on the carcass length. Moreover, an inspection of SSC17 which contained regions (i.e. cluster 14, 15 and 19) highly associated to CRCL from D1, joint and meta-analysis revealed genes potentially influencing this trait of interest: *PLCB1* (17:17,289,982–17,745,321, positive regulation of developmental growth GO: 0048639), *FLRT3* (17:22,940,499–22,954,452, embryonic morphogenesis GO: 0048598) and *FERMT1* (17:15,080,587–15,137,726, positive regulation of transforming growth factor beta receptor signaling pathway GO:0030511). Nevertheless, the most interesting finding was situated close to the highest significant SNP for CRCL in D1 (MARC0070553 with—log10 (*p* value) = 28.68 and *q* value = 9.25e-25) and is represented by *BMP2* (17:15,749,836–15,761,194). The bone morphogenetic protein 2 (*BMP2*) belongs to the same family as *BMP5* and is involved in the transforming growth factor-beta (TGF-beta) signaling pathway, playing a role in bone and cartilage development.

## Conclusion

A genome-wide association study was conducted for growth and carcass traits using SNP-chip information from three populations sharing a common founder breed (Piétrain). An individual population GWAS was conducted and two strategies for combining the datasets were employed: a joint population GWAS and a meta-analysis of the individual population GWAS summary statistics. While the joint population GWAS and the meta-analysis did not identify new associated regions besides the ones identified in the individual populations, both approaches had a positive impact on the mapping resolution which implies that causative mutations can be identified with higher accuracy. Depending on the access to the complete original datasets, the strategies can complement or substitute each other. A total of 20 genomic clusters were pinpointed and they contained genes previously associated with the traits (e.g. *IGF2*, *VRTN* and *TGFB3*). Finally, among the additional candidate genes being suggested, *BMP2* is being proposed as a strong candidate gene for carcass length. The findings of this study provide novel insights into approaches of dissecting the genetic basis of growth and carcass traits and indicate directions of further research which will lead to the identification of causal mutations affecting traits relevant in pig breeding programs.

## Supporting information

S1 Fig Overlap of identified clusters on SSC2, SSC7 and SSC17. (DOCX)

S1 Table List of significant SNPs at *p*_*nominal*_ ≤ 5*x*10^−5^, chromosomal position (bp) and cluster assignment for ADG, BFT, MFR and CRCL. The genome-wide significance threshold—log_10_ (*p*_*genome-wide*_) was 5.95 for D1, 5.91 for D2, 5.87 for D3, 5.81 for D1D2D3 and for MA. The nominal significance threshold was—log_10_ (*p*_*nominal*_) = 4.30. Genome-wide significant SNPs (*p*_*genome-wide*_ < 0.05) are written in boldface.
(DOCX)

S2 Table Pearson’s product-moment correlation for *p* values of the common markers in D1, D2, D3, D1D2D3 GWAS output and MA output. All significant at *p* < 0.05, except for the ones marked as ns = not significant.
(DOCX)
