# Supplementary material for: Genome-wide association studies and meta-analysis uncovers new candidate genes for growth and carcass traits in pigs
Source: PLoS One. 2018 Oct 11;13(10):e0205576. doi: 10.1371/journal.pone.0205576 (PMC6181390; doi:10.1371/journal.pone.0205576)
Supplement: S1 Fig — (DOCX) [file pone.0205576.s001.docx]

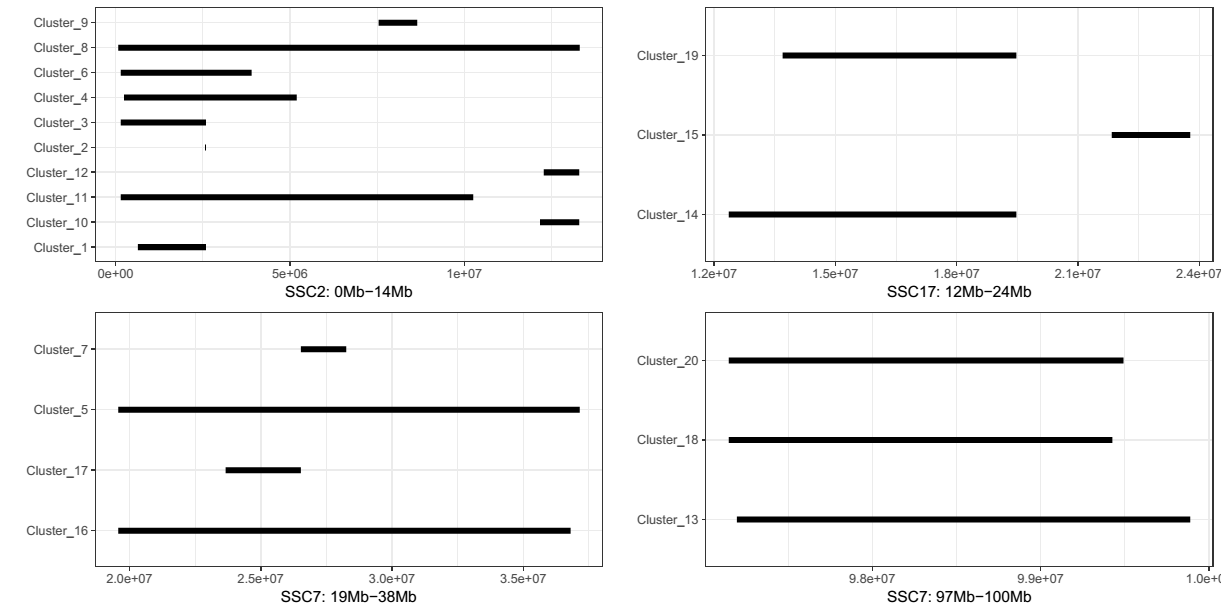


**S1 Fig**. **Overlap of identified clusters on SSC2, SSC7 and SSC17.**

**Reference**

**Ginestet C. ggplot2: Elegant Graphics for Data Analysis. J R Stat Soc a Stat. 2011;174:245-. doi: DOI 10.1111/j.1467-985X.2010.00676_9.x. PubMed PMID: WOS:000285969600026.**
