# Supplementary material for: Genome-wide association studies and meta-analysis uncovers new candidate genes for growth and carcass traits in pigs
Source: PLoS One. 2018 Oct 11;13(10):e0205576. doi: 10.1371/journal.pone.0205576 (PMC6181390; doi:10.1371/journal.pone.0205576)
Supplement: S1 Table — The genome-wide significance threshold—log10 (pgenome-wide) was 5.95 for D1, 5.91 for D2, 5.87 for D3, 5.81 for D1D2D3 and for MA. The nominal significance threshold was—log10 (pnominal) = 4.30. Genome-wide significant SNPs (pgenome-wide < 0.05) are written in boldface. (DOCX) [file pone.0205576.s002.docx]

**S1 Table. List of significant SNPs at** $\boldsymbol{p}_{\boldsymbol{nominal}}\boldsymbol{\leq5}\boldsymbol{x}\boldsymbol{10}^{\boldsymbol{-5}}$**, chromosomal position (bp) and cluster assignment for ADG, BFT, MFR and CRCL*.***

The genome-wide significance threshold –log_10_ ($p_{genome-wide}$) was 5.95 for D1, 5.91 for D2, 5.87 for D3, 5.81 for D1D2D3 and for MA. The nominal significance threshold was –log_10_ ($p_{nominal}$) = 4.30. Genome-wide significant SNPs ($p_{genome-wide}<0.05$) are written in boldface.

| Trait | Design | SSC | SNP | Pos (bp) | Cluster | -log10(p) |
| --- | --- | --- | --- | --- | --- | --- |
| ADG | D1 | 2 | ALGA0111915 | 141798 | - | 5,68140665 |
|  |  | 2 | ASGA0085784 | 236179 | - | 4,7249168 |
|  |  | 2 | ALGA0105438 | 631324 | 1 | **7,00351722** |
|  |  | 2 | ASGA0084177 | 677666 | 1 | **6,85499327** |
|  |  | 2 | ASGA0095646 | 750310 | 1 | 5,84889688 |
|  |  | 2 | ASGA0096364 | 1010999 | 1 | **6,09417175** |
|  |  | 2 | ALGA0123907 | 2556939 | 1 | **7,25694593** |
|  |  | 2 | M1GA0024408 | 2563214 | 1 | **6,70829157** |
|  |  | 2 | ASGA0102090 | 2586096 | 1 | 5,68036935 |
|  |  | 7 | ASGA0031928 | 22682744 | - | 5,38198435 |
|  |  | 7 | ALGA0039615 | 23177023 | - | **6,10742293** |
|  |  | 7 | ALGA0039819 | 25591627 | - | 5,19128141 |
|  |  | 7 | H3GA0020537 | 26083218 | - | 4,75472675 |
|  |  | 7 | H3GA0020700 | 29397842 | - | 4,94604616 |
|  |  | 13 | ASGA0098605 | 203230176 | - | 4,33774662 |
|  |  | 16 | H3GA0053033 | 18490106 | - | 5,04208685 |
| ADG | D3 | 8 | H3GA0024295 | 11805802 |  | 4,47313756 |
| ADG | D1D2D3 | 2 | ALGA0111915 | 141798 | - | 5,49511203 |
|  |  | 2 | ASGA0095646 | 750310 | - | 5,50635517 |
|  |  | 2 | ALGA0123907 | 2556939 | 2 | **6,85015608** |
|  |  | 2 | M1GA0024408 | 2563214 | 2 | **6,84320258** |
|  |  | 2 | ASGA0102090 | 2586096 | 2 | **6,22867273** |
|  |  | 7 | ALGA0039615 | 23177023 | - | 4,57081316 |
|  |  | 7 | ALGA0041945 | 50217275 | - | 4,38870908 |
|  |  | 8 | DIAS0003188 | 10755993 | - | 4,37320709 |
| ADG | MA | 2 | ALGA0111915 | 141798 | 3 | **6,85542579** |
|  |  | 2 | ASGA0095646 | 750310 | 3 | **6,93892468** |
|  |  | 2 | ASGA0085597 | 1083343 | 3 | 4,83268267 |
|  |  | 2 | M1GA0024950 | 2149306 | 3 | 4,88773023 |
|  |  | 2 | ALGA0106264 | 2380040 | 3 | 5,78914663 |
|  |  | 2 | MARC0044928 | 2494326 | 3 | 5,08181234 |
|  |  | 2 | ALGA0123907 | 2556939 | 3 | **8,15832775** |
|  |  | 2 | M1GA0024408 | 2563214 | 3 | **7,88206616** |
|  |  | 2 | ASGA0102090 | 2586096 | 3 | **7,49894074** |
|  |  | 2 | ASGA0008415 | 3895569 | - | 4,51798442 |
|  |  | 7 | ALGA0039615 | 23177023 | - | 5,65462627 |
| BFT | D1 | 1 | ASGA0099258 | 270868226 | - | 4,41553873 |
|  |  | 1 | MARC0097624 | 271131653 | - | 4,57302541 |
|  |  | 1 | ALGA0102621 | 274049382 | - | 4,75336603 |
|  |  | 2 | ASGA0084103 | 70140 | - | 4,33577372 |
|  |  | 2 | ALGA0111915 | 141798 | - | 5,47761576 |
|  |  | 2 | ASGA0085784 | 236179 | 4 | **8,08476484** |
|  |  | 2 | ALGA0110785 | 422658 | 4 | **10,6536646** |
|  |  | 2 | ALGA0105438 | 631324 | 4 | 5,32287871 |
|  |  | 2 | ASGA0084177 | 677666 | 4 | 4,98221148 |
|  |  | 2 | ASGA0095646 | 750310 | 4 | **7,12999428** |
|  |  | 2 | ASGA0096364 | 1010999 | 4 | 5,36762573 |
|  |  | 2 | ASGA0085597 | 1083343 | 4 | **10,7083049** |
|  |  | 2 | H3GA0054053 | 1100354 | 4 | **10,7083049** |
|  |  | 2 | MARC0113696 | 1318445 | 4 | 5,89833539 |
|  |  | 2 | M1GA0027267 | 1631678 | 4 | **7,94509981** |
|  |  | 2 | MARC0053928 | 1786591 | 4 | **7,00914722** |
|  |  | 2 | ASGA0105167 | 1925822 | 4 | 5,2150099 |
|  |  | 2 | M1GA0024950 | 2149306 | 4 | **7,2641489** |
|  |  | 2 | ALGA0106264 | 2380040 | 4 | **9,96262929** |
|  |  | 2 | MARC0044928 | 2494326 | 4 | **10,1396878** |
|  |  | 2 | ASGA0102090 | 2586096 | 4 | 4,88306035 |
|  |  | 2 | MARC0022036 | 2636399 | 4 | **6,02513245** |
|  |  | 2 | M1GA0025119 | 2694449 | 4 | **7,18920313** |
|  |  | 2 | MARC0095814 | 2697634 | 4 | **7,18920313** |
|  |  | 2 | H3GA0005490 | 2850536 | 4 | **7,5564554** |
|  |  | 2 | ASGA0086466 | 2938900 | 4 | 5,78937119 |
|  |  | 2 | MARC0008125 | 2984595 | 4 | **7,60746308** |
|  |  | 2 | ASGA0082213 | 3058654 | 4 | **7,54716669** |
|  |  | 2 | ASGA0083230 | 3062948 | 4 | **7,54716669** |
|  |  | 2 | ALGA0112642 | 3094550 | 4 | **7,41306967** |
|  |  | 2 | ASGA0008415 | 3895569 | 4 | **9,49003175** |
|  |  | 2 | H3GA0005547 | 4067805 | 4 | 5,60216771 |
|  |  | 2 | M1GA0002246 | 4365346 | 4 | **7,04492396** |
|  |  | 2 | H3GA0005584 | 4378975 | 4 | **7,47899536** |
|  |  | 2 | H3GA0005590 | 4412670 | 4 | 4,53621896 |
|  |  | 2 | ASGA0008564 | 5189397 | 4 | **7,3077587** |
|  |  | 2 | ASGA0102450 | 7449414 | - | 5,27450572 |
|  |  | 2 | H3GA0005759 | 7536991 | - | 4,86707145 |
|  |  | 2 | ASGA0008817 | 7877402 | - | 5,02637161 |
|  |  | 2 | ALGA0011706 | 8015941 | - | 5,02637161 |
|  |  | 2 | MARC0089099 | 8368989 | - | 5,8607886 |
|  |  | 2 | ALGA0112586 | 8371614 | - | 4,90409176 |
|  |  | 2 | H3GA0005861 | 8647689 | - | 4,45437254 |
|  |  | 2 | ALGA0011827 | 10254380 | - | 4,68166532 |
|  |  | 2 | MARC0082638 | 11467644 | - | 4,52236513 |
|  |  | 2 | ALGA0106245 | 12275048 | - | 4,32960106 |
|  |  | 10 | MARC0018305 | 16008154 | - | 4,44454762 |
|  |  | 10 | MARC0006021 | 16788025 | - | 4,61924442 |
| BFT | D2 | 5 | ASGA0091138 | 81631230 | - | 4,4509538 |
|  |  | 7 | M1GA0009653 | 16514926 | - | 5,22913768 |
|  |  | 7 | MARC0080224 | 16677064 | - | 5,51714499 |
|  |  | 7 | ALGA0039041 | 17305644 | - | 4,61521663 |
|  |  | 7 | DRGA0007264 | 17988475 | - | 4,38469609 |
|  |  | 7 | ALGA0039124 | 18172398 | - | 4,38469609 |
|  |  | 7 | DRGA0007280 | 18765001 | - | 4,3693098 |
|  |  | 7 | ASGA0031604 | 18948675 | - | 4,66014327 |
|  |  | 7 | ASGA0031662 | 19567933 | 5 | **8,86384407** |
|  |  | 7 | INRA0024039 | 19671985 | 5 | **8,86384407** |
|  |  | 7 | MARC0003822 | 19763746 | 5 | **6,20179907** |
|  |  | 7 | ASGA0083507 | 19971741 | 5 | 5,75436644 |
|  |  | 7 | DRGA0007316 | 20219344 | 5 | 4,51916552 |
|  |  | 7 | ASGA0031750 | 20235576 | 5 | 5,34937392 |
|  |  | 7 | H3GA0020255 | 20299952 | 5 | **6,23984074** |
|  |  | 7 | DRGA0007323 | 20329097 | 5 | 4,51916552 |
|  |  | 7 | ALGA0039319 | 20363677 | 5 | **6,23984074** |
|  |  | 7 | ALGA0039349 | 20657609 | 5 | 4,50935338 |
|  |  | 7 | ASGA0031780 | 20711029 | 5 | 4,50935338 |
|  |  | 7 | ALGA0039357 | 20736312 | 5 | 4,50935338 |
|  |  | 7 | MARC0056863 | 20805739 | 5 | 5,60690586 |
|  |  | 7 | M1GA0009695 | 20873545 | 5 | 4,94727913 |
|  |  | 7 | ASGA0031783 | 20918856 | 5 | 4,91138202 |
|  |  | 7 | MARC0059025 | 20980036 | 5 | 4,88646775 |
|  |  | 7 | ASGA0031806 | 20987924 | 5 | 5,23583284 |
|  |  | 7 | DIAS0002244 | 21001509 | 5 | 4,88646775 |
|  |  | 7 | ASGA0031822 | 21349121 | 5 | 5,71559408 |
|  |  | 7 | ALGA0039404 | 21366193 | 5 | **10,9735485** |
|  |  | 7 | ALGA0039405 | 21385114 | 5 | **10,9735485** |
|  |  | 7 | ASGA0031847 | 21673794 | 5 | 4,79748171 |
|  |  | 7 | ALGA0039447 | 21940478 | 5 | **10,9735485** |
|  |  | 7 | ALGA0039452 | 22008244 | 5 | 4,79748171 |
|  |  | 7 | ALGA0039480 | 22184943 | 5 | 4,35223181 |
|  |  | 7 | MARC0059955 | 22279830 | 5 | 5,23583284 |
|  |  | 7 | ALGA0039611 | 23037876 | 5 | **6,22524734** |
|  |  | 7 | MARC0091468 | 23110906 | 5 | **6,26411699** |
|  |  | 7 | ALGA0039615 | 23177023 | 5 | 4,7069138 |
|  |  | 7 | ALGA0039634 | 23504078 | 5 | 4,4125169 |
|  |  | 7 | H3GA0020462 | 24141402 | 5 | 4,77742138 |
|  |  | 7 | MARC0089437 | 24217931 | 5 | **10,3595424** |
|  |  | 7 | M1GA0009803 | 24259597 | 5 | 5,30036256 |
|  |  | 7 | MARC0024470 | 24296032 | 5 | 5,18447604 |
|  |  | 7 | MARC0058875 | 24865366 | 5 | **5,98734412** |
|  |  | 7 | ASGA0032099 | 24944510 | 5 | 4,78614769 |
|  |  | 7 | ALGA0039771 | 25026323 | 5 | **6,78542105** |
|  |  | 7 | H3GA0020505 | 25049757 | 5 | **6,01437506** |
|  |  | 7 | ASGA0032123 | 25400951 | 5 | **7,93795605** |
|  |  | 7 | ASGA0032135 | 25489746 | 5 | **8,50737509** |
|  |  | 7 | ALGA0039804 | 25516781 | 5 | **8,92420859** |
|  |  | 7 | DRGA0007396 | 25565917 | 5 | **11,9556445** |
|  |  | 7 | ALGA0039819 | 25591627 | 5 | **8,50737509** |
|  |  | 7 | ALGA0039826 | 25692312 | 5 | **8,50737509** |
|  |  | 7 | ALGA0039828 | 25698413 | 5 | **8,50737509** |
|  |  | 7 | MARC0087333 | 25830498 | 5 | **8,92564442** |
|  |  | 7 | ALGA0039842 | 25872510 | 5 | **8,92564442** |
|  |  | 7 | ASGA0032151 | 25967157 | 5 | 5,29614999 |
|  |  | 7 | DRGA0007412 | 26002978 | 5 | 4,52911196 |
|  |  | 7 | DRGA0007413 | 26015939 | 5 | **10,1003879** |
|  |  | 7 | INRA0024524 | 26069284 | 5 | **13,5620117** |
|  |  | 7 | DRGA0007416 | 26149917 | 5 | 5,7557798 |
|  |  | 7 | H3GA0020554 | 26394738 | 5 | **7,32091659** |
|  |  | 7 | ASGA0032174 | 26433052 | 5 | **9,41517256** |
|  |  | 7 | ALGA0039866 | 26451150 | 5 | 5,64086488 |
|  |  | 7 | BGIS0008247 | 26522116 | 5 | 5,65173019 |
|  |  | 7 | DRGA0007448 | 27134843 | 5 | 4,30326017 |
|  |  | 7 | MARC0050857 | 27284957 | 5 | 4,30326017 |
|  |  | 7 | ASGA0032239 | 27371495 | 5 | **8,54428438** |
|  |  | 7 | H3GA0020604 | 27394424 | 5 | 4,30326017 |
|  |  | 7 | ASGA0100868 | 27487930 | 5 | 4,30326017 |
|  |  | 7 | ALGA0115197 | 27504942 | 5 | 4,30326017 |
|  |  | 7 | MARC0098266 | 27519266 | 5 | 4,30326017 |
|  |  | 7 | SIRI0000698 | 27549577 | 5 | 4,30326017 |
|  |  | 7 | ASGA0032245 | 27562670 | 5 | 4,30326017 |
|  |  | 7 | ALGA0039998 | 27670833 | 5 | 4,30326017 |
|  |  | 7 | ALGA0040000 | 27707775 | 5 | 5,79570091 |
|  |  | 7 | ASGA0032255 | 27765368 | 5 | **8,54428438** |
|  |  | 7 | MARC0050171 | 27789010 | 5 | 4,30326017 |
|  |  | 7 | H3GA0020614 | 27865125 | 5 | 4,30326017 |
|  |  | 7 | ASGA0032257 | 27905935 | 5 | **8,54428438** |
|  |  | 7 | M1GA0009851 | 28029699 | 5 | **10,6150448** |
|  |  | 7 | DRGA0007462 | 28072350 | 5 | **8,54428438** |
|  |  | 7 | ASGA0032266 | 28115845 | 5 | **9,07132922** |
|  |  | 7 | M1GA0009853 | 28212506 | 5 | **8,97506713** |
|  |  | 7 | ASGA0032282 | 28252780 | 5 | **10,9530096** |
|  |  | 7 | H3GA0020623 | 28383120 | 5 | **8,82735621** |
|  |  | 7 | MARC0063300 | 28451792 | 5 | **10,9530096** |
|  |  | 7 | H3GA0020641 | 28521421 | 5 | **9,07132922** |
|  |  | 7 | ASGA0032304 | 28541774 | 5 | **9,07132922** |
|  |  | 7 | ALGA0040052 | 28559177 | 5 | **6,5917753** |
|  |  | 7 | ASGA0032313 | 28625619 | 5 | **9,07132922** |
|  |  | 7 | ASGA0032316 | 28655399 | 5 | 5,8151241 |
|  |  | 7 | DRGA0007475 | 28682123 | 5 | 4,5999942 |
|  |  | 7 | ALGA0040066 | 28721664 | 5 | 5,8151241 |
|  |  | 7 | ASGA0032320 | 28744265 | 5 | **10,9530096** |
|  |  | 7 | ASGA0032322 | 28798510 | 5 | **8,82735621** |
|  |  | 7 | ALGA0040076 | 28837944 | 5 | **9,07132922** |
|  |  | 7 | M1GA0009865 | 28897345 | 5 | **10,9530096** |
|  |  | 7 | ALGA0040085 | 28954803 | 5 | **9,07132922** |
|  |  | 7 | ALGA0119762 | 28998392 | 5 | **9,07132922** |
|  |  | 7 | ALGA0109063 | 29005790 | 5 | **9,07132922** |
|  |  | 7 | MARC0012864 | 29006040 | 5 | **8,65834789** |
|  |  | 7 | ASGA0032336 | 29060950 | 5 | **6,5917753** |
|  |  | 7 | ASGA0032342 | 29107207 | 5 | **11,3413229** |
|  |  | 7 | ALGA0040094 | 29122742 | 5 | **8,82735621** |
|  |  | 7 | MARC0068518 | 29166478 | 5 | **11,3413229** |
|  |  | 7 | ALGA0040113 | 29197718 | 5 | **11,3413229** |
|  |  | 7 | SIRI0000873 | 29228734 | 5 | **9,07132922** |
|  |  | 7 | M1GA0009879 | 29268803 | 5 | **11,3413229** |
|  |  | 7 | ALGA0040120 | 29289201 | 5 | **9,43689048** |
|  |  | 7 | MARC0079017 | 29372986 | 5 | **11,5816921** |
|  |  | 7 | ASGA0032398 | 29422512 | 5 | **8,13384799** |
|  |  | 7 | ALGA0040139 | 29442694 | 5 | **8,13384799** |
|  |  | 7 | ALGA0040140 | 29462756 | 5 | 5,14141348 |
|  |  | 7 | ALGA0040148 | 29487703 | 5 | **8,13384799** |
|  |  | 7 | ALGA0040153 | 29522461 | 5 | **8,13384799** |
|  |  | 7 | M1GA0009900 | 29638615 | 5 | 4,87393031 |
|  |  | 7 | ASGA0032422 | 29685247 | 5 | 5,13540646 |
|  |  | 7 | H3GA0020718 | 29707157 | 5 | **8,50044487** |
|  |  | 7 | M1GA0009910 | 29744642 | 5 | **8,20263273** |
|  |  | 7 | SIRI0000046 | 29878705 | 5 | 5,14141348 |
|  |  | 7 | ALGA0040227 | 30176520 | 5 | **6,74422111** |
|  |  | 7 | ALGA0040243 | 30213771 | 5 | 5,05440932 |
|  |  | 7 | MARC0033464 | 30572315 | 5 | **8,24885182** |
|  |  | 7 | ALGA0040298 | 30893735 | 5 | 4,9507781 |
|  |  | 7 | H3GA0020846 | 31235549 | 5 | **6,1107067** |
|  |  | 7 | H3GA0020852 | 31400744 | 5 | **9,29307408** |
|  |  | 7 | ASGA0032595 | 31569645 | 5 | 4,85979689 |
|  |  | 7 | H3GA0020876 | 32023608 | 5 | 4,5576943 |
|  |  | 7 | ASGA0032622 | 32054693 | 5 | 4,48097311 |
|  |  | 7 | DIAS0004649 | 32209256 | 5 | 4,7382567 |
|  |  | 7 | MARC0083062 | 32294915 | 5 | 4,42083972 |
|  |  | 7 | ASGA0032655 | 32492111 | 5 | **6,2068119** |
|  |  | 7 | M1GA0010037 | 32715205 | 5 | **6,84523834** |
|  |  | 7 | ALGA0040423 | 32827483 | 5 | **10,885716** |
|  |  | 7 | MARC0008120 | 32908115 | 5 | 4,76783772 |
|  |  | 7 | ALGA0037853 | 33003678 | 5 | 4,76783772 |
|  |  | 7 | ASGA0032683 | 33045445 | 5 | 4,76783772 |
|  |  | 7 | ALGA0040427 | 33062600 | 5 | 4,88904348 |
|  |  | 7 | MARC0014933 | 33216744 | 5 | 4,98297501 |
|  |  | 7 | M1GA0010050 | 33259678 | 5 | 4,53461566 |
|  |  | 7 | MARC0061348 | 33309304 | 5 | 4,53461566 |
|  |  | 7 | INRA0024930 | 34123154 | 5 | 4,85252773 |
|  |  | 7 | ALGA0040524 | 34151758 | 5 | 4,85252773 |
|  |  | 7 | DIAS0000554 | 34166932 | 5 | **13,1169697** |
|  |  | 7 | ASGA0032785 | 34422959 | 5 | 4,91840789 |
|  |  | 7 | M1GA0010080 | 34732894 | 5 | 5,07159851 |
|  |  | 7 | ALGA0040598 | 34772625 | 5 | **8,60679345** |
|  |  | 7 | MARC0073134 | 34786446 | 5 | **12,0235533** |
|  |  | 7 | ASGA0032847 | 34835490 | 5 | **8,01148296** |
|  |  | 7 | H3GA0021033 | 34899140 | 5 | 5,49482695 |
|  |  | 7 | H3GA0021027 | 34969376 | 5 | 4,40140316 |
|  |  | 7 | MARC0051108 | 35135602 | 5 | **7,97364654** |
|  |  | 7 | MARC0044921 | 35135926 | 5 | **11,3749253** |
|  |  | 7 | ALGA0040640 | 35268388 | 5 | **7,86077284** |
|  |  | 7 | INRA0025056 | 35394560 | 5 | **7,2313582** |
|  |  | 7 | ALGA0040695 | 35641882 | 5 | **8,11029165** |
|  |  | 7 | ASGA0032963 | 35667542 | 5 | **6,2979229** |
|  |  | 7 | ALGA0040717 | 35670068 | 5 | **8,11029165** |
|  |  | 7 | H3GA0021114 | 35747117 | 5 | **7,18756195** |
|  |  | 7 | ALGA0040721 | 35785388 | 5 | 5,7758452 |
|  |  | 7 | ALGA0040739 | 35970410 | 5 | **6,73872285** |
|  |  | 7 | ALGA0040759 | 36161073 | 5 | **6,9247524** |
|  |  | 7 | ALGA0040772 | 36203824 | 5 | **5,91370781** |
|  |  | 7 | ALGA0040777 | 36323988 | 5 | **6,72903018** |
|  |  | 7 | INRA0025107 | 36446715 | 5 | **8,11290169** |
|  |  | 7 | H3GA0021153 | 36465438 | 5 | 5,37045279 |
|  |  | 7 | MARC0060135 | 36587451 | 5 | **7,23868647** |
|  |  | 7 | ASGA0033035 | 36614274 | 5 | **6,12321887** |
|  |  | 7 | H3GA0021169 | 36646340 | 5 | 4,59084491 |
|  |  | 7 | MARC0066246 | 36707070 | 5 | 4,53574898 |
|  |  | 7 | ALGA0040805 | 36775483 | 5 | 4,39035544 |
|  |  | 7 | H3GA0021185 | 36795710 | 5 | **6,95989236** |
|  |  | 7 | H3GA0021205 | 37145252 | 5 | **6,12321887** |
|  |  | 7 | ASGA0033093 | 37331233 | - | 4,53993698 |
|  |  | 7 | DBMA0000241 | 39303646 | - | 4,81032164 |
|  |  | 7 | ALGA0040937 | 39316386 | - | 4,81032164 |
|  |  | 7 | ALGA0041064 | 41243726 | - | 5,38787762 |
|  |  | 7 | ASGA0033347 | 41971593 | - | 4,97502613 |
|  |  | 13 | MARC0080097 | 96537378 |  | **9,07132922** |
| BFT | D3 | 1 | ASGA0004792 | 147031524 | - | 4,31521027 |
|  |  | 2 | ASGA0008705 | 6884144 | - | 4,7540853 |
|  |  | 2 | DBWU0000981 | 7158021 | - | 4,87257122 |
|  |  | 2 | ALGA0114758 | 7363056 | - | 4,54644164 |
|  |  | 2 | ALGA0011643 | 7557050 | - | 4,96065728 |
|  |  | 2 | MARC0101520 | 7642626 | - | 4,77905291 |
|  |  | 2 | H3GA0005798 | 7751749 | - | 4,40870494 |
|  |  | 2 | ASGA0008809 | 7778736 | - | 4,98969156 |
|  |  | 4 | MARC0000845 | 79331946 | - | 4,63123909 |
|  |  | 4 | INRA0015172 | 79915989 | - | 5,46781656 |
| BFT | D1D2D3 | 1 | ASGA0099258 | 270868226 | - | 4,6585813 |
|  |  | 1 | H3GA0005295 | 272378806 | - | 4,32992122 |
|  |  | 2 | ALGA0111915 | 141798 | 6 | **7,58084422** |
|  |  | 2 | ALGA0110785 | 422658 | 6 | **11,7929287** |
|  |  | 2 | ASGA0095646 | 750310 | 6 | **5,95607606** |
|  |  | 2 | ASGA0085597 | 1083343 | 6 | **12,1414622** |
|  |  | 2 | M1GA0027267 | 1631678 | 6 | **8,92531153** |
|  |  | 2 | MARC0053928 | 1786591 | 6 | **6,42560986** |
|  |  | 2 | ALGA0106264 | 2380040 | 6 | **8,84792886** |
|  |  | 2 | MARC0044928 | 2494326 | 6 | **8,4484012** |
|  |  | 2 | MARC0095814 | 2697634 | 6 | **9,21346862** |
|  |  | 2 | H3GA0005490 | 2850536 | 6 | **9,62830056** |
|  |  | 2 | MARC0008125 | 2984595 | 6 | **8,92661529** |
|  |  | 2 | ASGA0083230 | 3062948 | 6 | **8,85608318** |
|  |  | 2 | ASGA0008415 | 3895569 | 6 | **11,9661663** |
|  |  | 2 | ASGA0102450 | 7449414 | - | 5,63820419 |
|  |  | 2 | ASGA0008817 | 7877402 | - | 4,51671089 |
|  |  | 2 | H3GA0005861 | 8647689 | - | 4,52338282 |
|  |  | 2 | ALGA0011827 | 10254380 | - | 4,74935315 |
|  |  | 7 | ALGA0039319 | 20363677 | - | 5,29910773 |
|  |  | 7 | MARC0089437 | 24217931 | - | **7,26343459** |
|  |  | 7 | BGIS0008247 | 26522116 | 7 | **5,99386355** |
|  |  | 7 | ASGA0032239 | 27371495 | 7 | 4,99817113 |
|  |  | 7 | M1GA0009851 | 28029699 | 7 | **6,62811237** |
|  |  | 7 | DRGA0007462 | 28072350 | 7 | 5,11738952 |
|  |  | 7 | ASGA0032282 | 28252780 | 7 | 4,93399852 |
|  |  | 7 | ASGA0032313 | 28625619 | - | 5,31984677 |
|  |  | 7 | ALGA0040085 | 28954803 | - | 5,19420224 |
|  |  | 7 | ALGA0109063 | 29005790 | - | 5,25264491 |
|  |  | 7 | MARC0012864 | 29006040 | - | 5,13319654 |
|  |  | 7 | MARC0068518 | 29166478 | - | **7,57472017** |
|  |  | 7 | ALGA0040120 | 29289201 | - | 5,57901341 |
|  |  | 7 | ALGA0040423 | 32827483 | - | 4,77476569 |
|  |  | 7 | M1GA0010050 | 33259678 | - | 4,65662095 |
| BFT | MA | 1 | ASGA0099258 | 270868226 | - | 4,62616885 |
|  |  | 1 | H3GA0005295 | 272378806 | - | 4,50501103 |
|  |  | 2 | ALGA0111915 | 141798 | 6 | **8,62727206** |
|  |  | 2 | ALGA0110785 | 422658 | 6 | **13,5377518** |
|  |  | 2 | ASGA0095646 | 750310 | 6 | **8,1632962** |
|  |  | 2 | ASGA0085597 | 1083343 | 6 | **13,7542406** |
|  |  | 2 | M1GA0027267 | 1631678 | 6 | **9,90483065** |
|  |  | 2 | MARC0053928 | 1786591 | 6 | **7,61439373** |
|  |  | 2 | ASGA0105167 | 1925822 | 6 | 4,45580789 |
|  |  | 2 | M1GA0024950 | 2149306 | 6 | **5,93181414** |
|  |  | 2 | ALGA0106264 | 2380040 | 6 | **10,3313346** |
|  |  | 2 | MARC0044928 | 2494326 | 6 | **10,4589202** |
|  |  | 2 | ASGA0102090 | 2586096 | 6 | 5,03044432 |
|  |  | 2 | MARC0022036 | 2636399 | 6 | **6,00682839** |
|  |  | 2 | MARC0095814 | 2697634 | 6 | **9,69723629** |
|  |  | 2 | H3GA0005490 | 2850536 | 6 | **10,8309136** |
|  |  | 2 | MARC0008125 | 2984595 | 6 | **10,3264262** |
|  |  | 2 | ASGA0083230 | 3062948 | 6 | **10,2659206** |
|  |  | 2 | ASGA0008415 | 3895569 | 6 | **14,0154727** |
|  |  | 2 | ALGA0011405 | 4134844 | - | 4,67695426 |
|  |  | 2 | DBWU0000981 | 7158021 | - | 5,36734029 |
|  |  | 2 | ASGA0102450 | 7449414 | - | **5,97715939** |
|  |  | 2 | ASGA0008817 | 7877402 | - | 4,32799455 |
|  |  | 2 | H3GA0005861 | 8647689 | - | 5,16851416 |
|  |  | 2 | ASGA0008974 | 10135131 | - | 4,66054856 |
|  |  | 2 | ALGA0011827 | 10254380 | - | 5,01139646 |
|  |  | 2 | ALGA0106245 | 12275048 | - | 4,53417118 |
|  |  | 7 | MARC0068518 | 29166478 | - | 4,71896663 |
| MFR | D1 | 1 | MARC0097624 | 271131653 | - | 5,90972784 |
|  |  | 1 | ALGA0010989 | 273570346 | - | 5,60653711 |
|  |  | 1 | ALGA0102621 | 274049382 | - | 4,71076567 |
|  |  | 2 | ASGA0084103 | 70140 | 8 | **8,90002947** |
|  |  | 2 | ALGA0111915 | 141798 | 8 | **17,3276906** |
|  |  | 2 | ASGA0106410 | 145285 | 8 | **9,78294266** |
|  |  | 2 | ASGA0085784 | 236179 | 8 | **18,7239244** |
|  |  | 2 | ASGA0084522 | 269252 | 8 | **6,64087438** |
|  |  | 2 | ASGA0097367 | 310804 | 8 | **9,78294266** |
|  |  | 2 | ALGA0110785 | 422658 | 8 | **19,9463915** |
|  |  | 2 | ALGA0105438 | 631324 | 8 | **18,4919203** |
|  |  | 2 | ASGA0084177 | 677666 | 8 | **16,3088248** |
|  |  | 2 | ASGA0095646 | 750310 | 8 | **17,4704426** |
|  |  | 2 | ASGA0096364 | 1010999 | 8 | **17,0356201** |
|  |  | 2 | ASGA0085597 | 1083343 | 8 | **19,8153001** |
|  |  | 2 | H3GA0054053 | 1100354 | 8 | **19,8153001** |
|  |  | 2 | MARC0113696 | 1318445 | 8 | **7,7451282** |
|  |  | 2 | M1GA0027267 | 1631678 | 8 | **13,2095354** |
|  |  | 2 | MARC0053928 | 1786591 | 8 | **14,3708555** |
|  |  | 2 | ASGA0105167 | 1925822 | 8 | **8,26962396** |
|  |  | 2 | M1GA0024950 | 2149306 | 8 | **14,2304395** |
|  |  | 2 | ALGA0106264 | 2380040 | 8 | **21,0610642** |
|  |  | 2 | MARC0044928 | 2494326 | 8 | **21,2837462** |
|  |  | 2 | ALGA0123907 | 2556939 | 8 | **10,4735819** |
|  |  | 2 | M1GA0024408 | 2563214 | 8 | **9,43670644** |
|  |  | 2 | ASGA0102090 | 2586096 | 8 | **14,1617909** |
|  |  | 2 | ASGA0098481 | 2594104 | 8 | **7,74739451** |
|  |  | 2 | MARC0022036 | 2636399 | 8 | **13,7131956** |
|  |  | 2 | M1GA0025119 | 2694449 | 8 | **14,5232365** |
|  |  | 2 | MARC0095814 | 2697634 | 8 | **14,5232365** |
|  |  | 2 | H3GA0005490 | 2850536 | 8 | **13,6388674** |
|  |  | 2 | ALGA0116344 | 2851936 | 8 | 4,59477149 |
|  |  | 2 | ALGA0103099 | 2878084 | 8 | **7,02980706** |
|  |  | 2 | ASGA0086466 | 2938900 | 8 | **9,79270502** |
|  |  | 2 | MARC0008125 | 2984595 | 8 | **13,4966997** |
|  |  | 2 | ASGA0082213 | 3058654 | 8 | **13,4322387** |
|  |  | 2 | ASGA0083230 | 3062948 | 8 | **13,4322387** |
|  |  | 2 | ALGA0112642 | 3094550 | 8 | **13,1045775** |
|  |  | 2 | DIAS0001270 | 3257622 | 8 | 5,39559357 |
|  |  | 2 | ASGA0008415 | 3895569 | 8 | **16,0270954** |
|  |  | 2 | H3GA0005547 | 4067805 | 8 | **10,6638886** |
|  |  | 2 | M1GA0002229 | 4341271 | 8 | 5,67288025 |
|  |  | 2 | M1GA0002246 | 4365346 | 8 | **11,6978185** |
|  |  | 2 | H3GA0005584 | 4378975 | 8 | **17,975305** |
|  |  | 2 | H3GA0005590 | 4412670 | 8 | **6,42889005** |
|  |  | 2 | ALGA0109310 | 4531334 | 8 | **8,07241819** |
|  |  | 2 | MARC0045154 | 4671904 | 8 | 4,53120679 |
|  |  | 2 | M1GA0002294 | 5002263 | 8 | **7,36641036** |
|  |  | 2 | ASGA0008564 | 5189397 | 8 | **11,69667** |
|  |  | 2 | DIAS0004764 | 5587612 | 8 | **6,19176471** |
|  |  | 2 | ASGA0102179 | 5707905 | 8 | 5,43144512 |
|  |  | 2 | MARC0089109 | 5900258 | 8 | **7,61594243** |
|  |  | 2 | ALGA0011510 | 5998495 | 8 | **7,58945726** |
|  |  | 2 | ALGA0112279 | 7302964 | 8 | **6,58171031** |
|  |  | 2 | ASGA0008769 | 7316443 | 8 | **6,88103132** |
|  |  | 2 | ASGA0102450 | 7449414 | 8 | 4,82718698 |
|  |  | 2 | H3GA0005759 | 7536991 | 8 | **7,98445583** |
|  |  | 2 | H3GA0005778 | 7680580 | 8 | **7,99846098** |
|  |  | 2 | ASGA0008817 | 7877402 | 8 | 5,44175957 |
|  |  | 2 | ALGA0011706 | 8015941 | 8 | 5,44175957 |
|  |  | 2 | MARC0089099 | 8368989 | 8 | **7,25807194** |
|  |  | 2 | ALGA0112586 | 8371614 | 8 | **6,58989623** |
|  |  | 2 | ASGA0008859 | 8518027 | 8 | 4,77057927 |
|  |  | 2 | ALGA0011728 | 8584094 | 8 | 4,6706245 |
|  |  | 2 | H3GA0005861 | 8647689 | 8 | 5,8198942 |
|  |  | 2 | H3GA0053137 | 9252505 | 8 | 4,85274426 |
|  |  | 2 | ASGA0008896 | 9614500 | 8 | 5,78103409 |
|  |  | 2 | ALGA0011827 | 10254380 | 8 | **6,76292398** |
|  |  | 2 | ALGA0011927 | 10752126 | 8 | 4,55943251 |
|  |  | 2 | ALGA0011860 | 11440433 | 8 | **9,13925425** |
|  |  | 2 | MARC0082638 | 11467644 | 8 | **10,2885024** |
|  |  | 2 | MARC0033827 | 11501570 | 8 | **8,53619806** |
|  |  | 2 | ALGA0109371 | 11623996 | 8 | **8,46620634** |
|  |  | 2 | ALGA0108682 | 11627275 | 8 | **8,46620634** |
|  |  | 2 | ALGA0119954 | 11683892 | 8 | **8,07933779** |
|  |  | 2 | MARC0065010 | 11693302 | 8 | **8,07933779** |
|  |  | 2 | ASGA0102837 | 11711252 | 8 | **6,04418626** |
|  |  | 2 | ASGA0101291 | 11716342 | 8 | **8,07933779** |
|  |  | 2 | MARC0090671 | 11745401 | 8 | 5,4332138 |
|  |  | 2 | MARC0046222 | 11975649 | 8 | 4,50791742 |
|  |  | 2 | ASGA0009119 | 12168412 | 8 | 5,47091373 |
|  |  | 2 | ALGA0106126 | 12210974 | 8 | 4,70649381 |
|  |  | 2 | ALGA0106245 | 12275048 | 8 | **6,29662091** |
|  |  | 2 | ASGA0009136 | 12484742 | 8 | **6,48318444** |
|  |  | 2 | ALGA0112610 | 12521316 | 8 | **6,48318444** |
|  |  | 2 | ALGA0119337 | 12643335 | 8 | **6,7758452** |
|  |  | 2 | ASGA0094290 | 12656003 | 8 | **6,7758452** |
|  |  | 2 | ASGA0105274 | 12725515 | 8 | **6,31215279** |
|  |  | 2 | ASGA0101621 | 12817770 | 8 | 4,80460963 |
|  |  | 2 | ALGA0113046 | 12842161 | 8 | **6,10820951** |
|  |  | 2 | ASGA0086549 | 12864431 | 8 | 5,93110574 |
|  |  | 2 | ASGA0094487 | 12865452 | 8 | **6,7758452** |
|  |  | 2 | H3GA0006033 | 13076786 | 8 | **6,61285409** |
|  |  | 2 | ASGA0009185 | 13105939 | 8 | **6,72026463** |
|  |  | 2 | UMB10000151 | 13156928 | 8 | 4,36554994 |
|  |  | 2 | DIAS0000317 | 13167684 | 8 | 4,36554994 |
|  |  | 2 | ALGA0012032 | 13192805 | 8 | **6,63584502** |
|  |  | 2 | ALGA0012034 | 13218267 | 8 | **6,63584502** |
|  |  | 2 | H3GA0006044 | 13230545 | 8 | **7,34022352** |
|  |  | 2 | ASGA0009211 | 13294789 | 8 | **6,63584502** |
|  |  | 2 | ALGA0012049 | 13307467 | 8 | **6,80355851** |
|  |  | 2 | ASGA0085457 | 13400592 | - | 4,75827924 |
|  |  | 2 | ASGA0096073 | 13442379 | - | 4,84979023 |
| MFR | D2 | 2 | MARC0066239 | 2036007 | - | 4,36805323 |
|  |  | 2 | ALGA0104042 | 2036226 | - | 4,52548945 |
|  |  | 2 | MARC0053324 | 2119793 | - | 4,36805323 |
|  |  | 2 | ASGA0098549 | 2375454 | - | 4,43448157 |
|  |  | 2 | MARC0095814 | 2697634 | - | 4,54216199 |
|  |  | 2 | ASGA0089068 | 3237229 | - | 5,35297028 |
|  |  | 12 | ALGA0123085 | 18043041 | - | 4,33934319 |
| MFR | D3 | 2 | M1GA0002346 | 6511747 | - | 4,57133562 |
|  |  | 2 | ASGA0008646 | 6525911 | - | 4,57133562 |
|  |  | 2 | ASGA0008649 | 6537999 | - | 4,57133562 |
|  |  | 2 | H3GA0005672 | 6689718 | - | 4,57133562 |
|  |  | 2 | ASGA0008662 | 6714052 | - | 4,57133562 |
|  |  | 2 | ASGA0008695 | 6823767 | - | 4,57133562 |
|  |  | 2 | DIAS0004469 | 7143618 | - | 5,17083651 |
|  |  | 2 | DBWU0000981 | 7158021 | - | 4,37579631 |
|  |  | 2 | ALGA0105044 | 7176922 | - | 4,30094836 |
|  |  | 2 | MARC0023230 | 7244783 | - | 5,17083651 |
|  |  | 2 | ASGA0008769 | 7316443 | - | 4,94877028 |
|  |  | 2 | CADI0000661 | 7420356 | - | 5,17083651 |
|  |  | 2 | DIAS0004174 | 7420356 | - | 5,17083651 |
|  |  | 2 | ALGA0011643 | 7557050 | - | 5,69636123 |
|  |  | 2 | ALGA0011652 | 7589272 | - | 5,29553117 |
| MFR | D1D2D3 | 1 | DRGA0001536 | 133513858 | - | 4,5997453 |
|  |  | 1 | M1GA0001857 | 271083624 | - | **6,42715743** |
|  |  | 1 | ASGA0008080 | 271105069 | - | 5,58605347 |
|  |  | 1 | H3GA0005295 | 272378806 | - | 4,81551015 |
|  |  | 1 | MARC0025058 | 272699190 | - | 4,66849375 |
|  |  | 2 | ALGA0111915 | 141798 | 6 | **18,1975916** |
|  |  | 2 | ALGA0110785 | 422658 | 6 | **20,7336436** |
|  |  | 2 | ASGA0095646 | 750310 | 6 | **13,75429** |
|  |  | 2 | ASGA0085597 | 1083343 | 6 | **20,8407818** |
|  |  | 2 | M1GA0027267 | 1631678 | 6 | **13,59718** |
|  |  | 2 | MARC0053928 | 1786591 | 6 | **10,0440295** |
|  |  | 2 | M1GA0024950 | 2149306 | 6 | **6,25838755** |
|  |  | 2 | ALGA0106264 | 2380040 | 6 | **15,4864468** |
|  |  | 2 | MARC0044928 | 2494326 | 6 | **14,3353524** |
|  |  | 2 | ALGA0123907 | 2556939 | 6 | 5,19255901 |
|  |  | 2 | M1GA0024408 | 2563214 | 6 | 5,30622734 |
|  |  | 2 | ASGA0102090 | 2586096 | 6 | **10,5303517** |
|  |  | 2 | MARC0022036 | 2636399 | 6 | **6,7491898** |
|  |  | 2 | MARC0095814 | 2697634 | 6 | **15,5000491** |
|  |  | 2 | H3GA0005490 | 2850536 | 6 | **15,1295762** |
|  |  | 2 | MARC0008125 | 2984595 | 6 | **14,6264683** |
|  |  | 2 | ASGA0083230 | 3062948 | 6 | **14,5674476** |
|  |  | 2 | ASGA0008415 | 3895569 | 6 | **15,4103553** |
|  |  | 2 | M1GA0002229 | 4341271 | - | 4,64918622 |
|  |  | 2 | M1GA0002294 | 5002263 | - | 4,46301252 |
|  |  | 2 | H3GA0005759 | 7536991 | 9 | 5,02296083 |
|  |  | 2 | H3GA0005778 | 7680580 | 9 | 4,93054651 |
|  |  | 2 | ASGA0008817 | 7877402 | 9 | 5,46041836 |
|  |  | 2 | MARC0089099 | 8368989 | 9 | **6,23571549** |
|  |  | 2 | ALGA0112586 | 8371614 | 9 | 5,66124876 |
|  |  | 2 | H3GA0005861 | 8647689 | 9 | **6,73844691** |
|  |  | 2 | ALGA0011827 | 10254380 | - | 5,72222581 |
|  |  | 2 | ASGA0009119 | 12168412 | 10 | 4,3050475 |
|  |  | 2 | ALGA0106126 | 12210974 | 10 | 4,31992206 |
|  |  | 2 | ALGA0106245 | 12275048 | 10 | 5,56996528 |
|  |  | 2 | ASGA0009136 | 12484742 | 10 | **6,20159384** |
|  |  | 2 | ALGA0112610 | 12521316 | 10 | **6,20159384** |
|  |  | 2 | ASGA0105274 | 12725515 | 10 | 4,64171291 |
|  |  | 2 | ALGA0113046 | 12842161 | 10 | 5,54942298 |
|  |  | 2 | ASGA0086549 | 12864431 | 10 | 5,38766868 |
|  |  | 2 | H3GA0006029 | 12935396 | 10 | **7,60859025** |
|  |  | 2 | H3GA0006033 | 13076786 | 10 | 5,59398299 |
|  |  | 2 | ASGA0009211 | 13294789 | 10 | 4,63332308 |
| MFR | MA | 1 | ASGA0099258 | 270868226 | - | 4,36713796 |
|  |  | 1 | M1GA0001857 | 271083624 | - | **6,06889825** |
|  |  | 1 | ASGA0008080 | 271105069 | - | 4,8256494 |
|  |  | 1 | H3GA0005257 | 271626303 | - | 4,85294233 |
|  |  | 1 | ALGA0010930 | 272001272 | - | 4,60519822 |
|  |  | 1 | H3GA0005295 | 272378806 | - | 5,16115091 |
|  |  | 2 | ALGA0111915 | 141798 | 11 | **20,9986991** |
|  |  | 2 | ALGA0110785 | 422658 | 11 | **23,9821323** |
|  |  | 2 | ASGA0095646 | 750310 | 11 | **18,8843895** |
|  |  | 2 | ASGA0085597 | 1083343 | 11 | **24,0379149** |
|  |  | 2 | M1GA0027267 | 1631678 | 11 | **16,0451306** |
|  |  | 2 | MARC0053928 | 1786591 | 11 | **15,1374512** |
|  |  | 2 | ASGA0105167 | 1925822 | 11 | **7,32790214** |
|  |  | 2 | M1GA0024950 | 2149306 | 11 | **12,5131446** |
|  |  | 2 | ALGA0106264 | 2380040 | 11 | **21,1773743** |
|  |  | 2 | MARC0044928 | 2494326 | 11 | **20,8416375** |
|  |  | 2 | ALGA0123907 | 2556939 | 11 | **8,30821148** |
|  |  | 2 | M1GA0024408 | 2563214 | 11 | **8,00331324** |
|  |  | 2 | ASGA0102090 | 2586096 | 11 | **13,9157813** |
|  |  | 2 | MARC0022036 | 2636399 | 11 | **12,3257821** |
|  |  | 2 | MARC0095814 | 2697634 | 11 | **17,1508264** |
|  |  | 2 | H3GA0005490 | 2850536 | 11 | **17,0234208** |
|  |  | 2 | MARC0008125 | 2984595 | 11 | **16,573001** |
|  |  | 2 | ASGA0083230 | 3062948 | 11 | **16,5077985** |
|  |  | 2 | H3GA0005515 | 3859973 | 11 | 4,7115272 |
|  |  | 2 | ASGA0008415 | 3895569 | 11 | **19,3178549** |
|  |  | 2 | ALGA0011405 | 4134844 | 11 | 5,33479437 |
|  |  | 2 | M1GA0002229 | 4341271 | 11 | **6,32284948** |
|  |  | 2 | H3GA0005590 | 4412670 | 11 | **6,02946717** |
|  |  | 2 | M1GA0002294 | 5002263 | 11 | **5,82885885** |
|  |  | 2 | MARC0089109 | 5900258 | 11 | **6,26656197** |
|  |  | 2 | ALGA0112279 | 7302964 | 11 | 4,67633546 |
|  |  | 2 | ASGA0102450 | 7449414 | 11 | 4,98046832 |
|  |  | 2 | H3GA0005759 | 7536991 | 11 | **6,3638131** |
|  |  | 2 | H3GA0005778 | 7680580 | 11 | **6,35684353** |
|  |  | 2 | ASGA0008817 | 7877402 | 11 | **6,20957408** |
|  |  | 2 | H3GA0005813 | 7987374 | 11 | 4,34823755 |
|  |  | 2 | H3GA0005831 | 8073828 | 11 | 4,39115318 |
|  |  | 2 | MARC0089099 | 8368989 | 11 | **6,69400412** |
|  |  | 2 | ALGA0112586 | 8371614 | 11 | **6,23017974** |
|  |  | 2 | H3GA0005861 | 8647689 | 11 | **7,98842956** |
|  |  | 2 | ASGA0008896 | 9614500 | 11 | 4,99012437 |
|  |  | 2 | ALGA0011827 | 10254380 | 11 | **6,92081875** |
|  |  | 2 | ASGA0009119 | 12168412 | - | 5,10551778 |
|  |  | 2 | ALGA0106126 | 12210974 | - | 4,79155864 |
|  |  | 2 | ALGA0106245 | 12275048 | 12 | **6,67039875** |
|  |  | 2 | ASGA0009136 | 12484742 | 12 | **6,66655273** |
|  |  | 2 | ALGA0112610 | 12521316 | 12 | **6,66655273** |
|  |  | 2 | ASGA0105274 | 12725515 | 12 | **6,13418562** |
|  |  | 2 | ALGA0113046 | 12842161 | 12 | **6,12482294** |
|  |  | 2 | ASGA0086549 | 12864431 | 12 | **5,97346674** |
|  |  | 2 | H3GA0006029 | 12935396 | 12 | 5,69637202 |
|  |  | 2 | H3GA0006033 | 13076786 | 12 | **6,50293206** |
|  |  | 2 | ASGA0009211 | 13294789 | 12 | **6,5055671** |
| CRCL | D1 | 7 | DIAS0001336 | 23406030 | - | 4,56511507 |
|  |  | 7 | ASGA0031989 | 23444442 | - | 4,61303934 |
|  |  | 7 | H3GA0020425 | 23532752 | - | 5,224169 |
|  |  | 7 | DIAS0004622 | 23659424 | - | 4,39886604 |
|  |  | 7 | SIRI0000267 | 87516360 | - | 5,70490814 |
|  |  | 7 | ASGA0034874 | 87543395 | - | 4,54217561 |
|  |  | 7 | H3GA0022644 | 97147161 | - | 5,24412591 |
|  |  | 7 | DRGA0008026 | 97195350 | 13 | **8,48112693** |
|  |  | 7 | ALGA0043941 | 97247184 | 13 | **8,48112693** |
|  |  | 7 | H3GA0022648 | 97279129 | 13 | **6,14147303** |
|  |  | 7 | INRA0027623 | 97521999 | 13 | **6,22130839** |
|  |  | 7 | M1GA0010653 | 97795647 | 13 | 4,47500316 |
|  |  | 7 | ASGA0035535 | 98186259 | 13 | 4,97528042 |
|  |  | 7 | ASGA0035536 | 98264173 | 13 | 4,97528042 |
|  |  | 7 | ALGA0043984 | 99116565 | 13 | 5,81266545 |
|  |  | 7 | ALGA0044022 | 99337831 | 13 | **8,82111798** |
|  |  | 7 | ASGA0035589 | 99414005 | 13 | **5,95343851** |
|  |  | 7 | ASGA0035590 | 99424987 | 13 | **6,10165993** |
|  |  | 7 | H3GA0022712 | 99491117 | 13 | **7,55443471** |
|  |  | 7 | ASGA0035609 | 99677153 | 13 | 5,20368035 |
|  |  | 7 | H3GA0022720 | 99705755 | 13 | **6,56074154** |
|  |  | 7 | H3GA0022724 | 99887568 | 13 | **7,9159137** |
|  |  | 7 | ALGA0044121 | 100545566 | - | 5,04979097 |
|  |  | 16 | H3GA0046250 | 22602483 | - | 4,38096827 |
|  |  | 17 | ALGA0092525 | 1066109 | - | 4,929903 |
|  |  | 17 | MARC0061426 | 1135064 | - | 4,929903 |
|  |  | 17 | ALGA0092584 | 1734092 | - | 4,36919698 |
|  |  | 17 | ASGA0074980 | 1971412 | - | 4,37266692 |
|  |  | 17 | ALGA0093074 | 11374094 | - | 4,31862759 |
|  |  | 17 | ALGA0093288 | 12101104 | - | 5,13385095 |
|  |  | 17 | ALGA0093254 | 12361530 | 14 | **6,10947783** |
|  |  | 17 | ASGA0075436 | 12416997 | 14 | **6,10947783** |
|  |  | 17 | H3GA0047937 | 13692477 | 14 | **7,73554818** |
|  |  | 17 | ALGA0093386 | 13877448 | 14 | 5,35598508 |
|  |  | 17 | ASGA0089601 | 13979012 | 14 | **6,83318816** |
|  |  | 17 | ALGA0103055 | 14252000 | 14 | **7,22644831** |
|  |  | 17 | MARC0066372 | 14439916 | 14 | 4,32951572 |
|  |  | 17 | ASGA0075536 | 15196027 | 14 | **9,19716814** |
|  |  | 17 | MARC0056509 | 15256964 | 14 | **14,8125272** |
|  |  | 17 | ALGA0093434 | 15321777 | 14 | **12,210767** |
|  |  | 17 | ASGA0075539 | 15389339 | 14 | **7,26307947** |
|  |  | 17 | ALGA0093437 | 15401211 | 14 | **11,6742873** |
|  |  | 17 | ALGA0093440 | 15421803 | 14 | **9,47747361** |
|  |  | 17 | INRA0052780 | 15659761 | 14 | **6,45612557** |
|  |  | 17 | MARC0112426 | 15755718 | 14 | 5,67992437 |
|  |  | 17 | MARC0074172 | 15768175 | 14 | **14,4158679** |
|  |  | 17 | MARC0070553 | 15827832 | 14 | **28,6816799** |
|  |  | 17 | INRA0052808 | 15896846 | 14 | **17,6727903** |
|  |  | 17 | CASI0008233 | 15904945 | 14 | 5,56127733 |
|  |  | 17 | ALGA0093449 | 15919300 | 14 | 5,7167689 |
|  |  | 17 | ALGA0093461 | 16279385 | 14 | 4,71559183 |
|  |  | 17 | ASGA0075556 | 16422085 | 14 | **11,0885004** |
|  |  | 17 | MARC0028591 | 16634318 | 14 | **6,87617122** |
|  |  | 17 | ALGA0105626 | 16828543 | 14 | **9,10584627** |
|  |  | 17 | ALGA0093478 | 16919581 | 14 | **21,732671** |
|  |  | 17 | ALGA0093481 | 16940859 | 14 | **9,70096364** |
|  |  | 17 | ALGA0093482 | 16966248 | 14 | **7,42552309** |
|  |  | 17 | ALGA0093484 | 17075321 | 14 | 4,91787195 |
|  |  | 17 | DRGA0016598 | 17258153 | 14 | 5,00579507 |
|  |  | 17 | MARC0027977 | 17667594 | 14 | **10,8794623** |
|  |  | 17 | ASGA0075596 | 17755511 | 14 | **10,8794623** |
|  |  | 17 | ALGA0093563 | 17867191 | 14 | 5,53490727 |
|  |  | 17 | INRA0052898 | 17910278 | 14 | 5,53490727 |
|  |  | 17 | ASGA0075611 | 17937033 | 14 | **9,073388** |
|  |  | 17 | INRA0052906 | 17954922 | 14 | 5,53490727 |
|  |  | 17 | ALGA0093570 | 18039052 | 14 | **7,21188044** |
|  |  | 17 | DRGA0016604 | 18070486 | 14 | 5,45605233 |
|  |  | 17 | H3GA0048016 | 18389848 | 14 | 5,1071366 |
|  |  | 17 | ALGA0093616 | 18543305 | 14 | **8,31325919** |
|  |  | 17 | MARC0091816 | 18565068 | 14 | 4,6558672 |
|  |  | 17 | ASGA0075671 | 18738724 | 14 | 4,43315845 |
|  |  | 17 | H3GA0048042 | 19474175 | 14 | **6,45181211** |
|  |  | 17 | ASGA0097904 | 19639240 | - | 4,76744857 |
|  |  | 17 | H3GA0048065 | 20221234 | - | 4,71381758 |
|  |  | 17 | MARC0080340 | 20310514 | - | 5,35346199 |
|  |  | 17 | MARC0034730 | 20418829 | - | 4,8879315 |
|  |  | 17 | ASGA0075714 | 20434043 | - | 4,65933185 |
|  |  | 17 | DRGA0016627 | 20677863 | - | 5,15927993 |
|  |  | 17 | DRGA0016629 | 20695024 | - | 5,41107426 |
|  |  | 17 | MARC0055684 | 20763022 | - | 5,39146648 |
|  |  | 17 | CASI0007925 | 20854664 | - | 5,72004803 |
|  |  | 17 | MARC0039322 | 20991979 | - | 5,72004803 |
|  |  | 17 | DRGA0016636 | 21244125 | - | 5,55663999 |
|  |  | 17 | MARC0039749 | 21832087 | 15 | **6,52539479** |
|  |  | 17 | H3GA0048092 | 21862282 | 15 | **6,10829532** |
|  |  | 17 | ALGA0093780 | 22108491 | 15 | **5,95034403** |
|  |  | 17 | ALGA0121594 | 22420488 | 15 | **6,11848924** |
|  |  | 17 | INRA0053098 | 22422487 | 15 | **6,11848924** |
|  |  | 17 | INRA0053108 | 22775310 | 15 | **6,11848924** |
|  |  | 17 | MARC0016887 | 22870401 | 15 | **11,853379** |
|  |  | 17 | ALGA0093828 | 23020782 | 15 | 4,39099715 |
|  |  | 17 | MARC0030185 | 23431948 | 15 | **6,14812124** |
|  |  | 17 | ALGA0093843 | 23687689 | 15 | **11,853379** |
|  |  | 17 | MARC0058896 | 23773788 | 15 | **7,87829574** |
|  |  | 17 | MARC0115548 | 25624531 | - | 5,60909667 |
|  |  | 17 | DRGA0016685 | 28172397 | - | 4,60172137 |
|  |  | 17 | ALGA0094134 | 28210436 | - | 4,85368278 |
|  |  | 17 | MARC0061206 | 28823286 | - | 5,93450618 |
| CRCL | D2 | 7 | M1GA0009653 | 16514926 | - | 4,44675797 |
|  |  | 7 | H3GA0020165 | 17931796 | - | 4,5374867 |
|  |  | 7 | ALGA0039105 | 17964972 | - | 4,52718068 |
|  |  | 7 | DRGA0007280 | 18765001 | - | 4,43976688 |
|  |  | 7 | ASGA0031614 | 19138092 | - | 4,67245513 |
|  |  | 7 | ASGA0031662 | 19567933 | 16 | **9,1040512** |
|  |  | 7 | ALGA0039211 | 19615474 | 16 | 5,73844453 |
|  |  | 7 | INRA0024039 | 19671985 | 16 | **9,1040512** |
|  |  | 7 | ASGA0031679 | 19710658 | 16 | 5,51867922 |
|  |  | 7 | MARC0003822 | 19763746 | 16 | **6,85034989** |
|  |  | 7 | DRGA0007316 | 20219344 | 16 | 5,07891207 |
|  |  | 7 | ASGA0031750 | 20235576 | 16 | 4,86395518 |
|  |  | 7 | H3GA0020255 | 20299952 | 16 | **8,11009071** |
|  |  | 7 | DRGA0007323 | 20329097 | 16 | 5,07891207 |
|  |  | 7 | ALGA0039319 | 20363677 | 16 | **8,11009071** |
|  |  | 7 | H3GA0020268 | 20399615 | 16 | 4,78675061 |
|  |  | 7 | ALGA0039341 | 20467978 | 16 | 5,35178933 |
|  |  | 7 | H3GA0020279 | 20498625 | 16 | 5,35178933 |
|  |  | 7 | MARC0073551 | 20557169 | 16 | 5,02916145 |
|  |  | 7 | ALGA0039349 | 20657609 | 16 | 5,8928545 |
|  |  | 7 | ASGA0031780 | 20711029 | 16 | 5,8928545 |
|  |  | 7 | ALGA0039357 | 20736312 | 16 | 5,8928545 |
|  |  | 7 | MARC0056863 | 20805739 | 16 | 5,65853778 |
|  |  | 7 | M1GA0009695 | 20873545 | 16 | **6,2410412** |
|  |  | 7 | ASGA0031783 | 20918856 | 16 | **5,96117327** |
|  |  | 7 | MARC0059025 | 20980036 | 16 | 5,89382611 |
|  |  | 7 | ASGA0031806 | 20987924 | 16 | 5,32056211 |
|  |  | 7 | DIAS0002244 | 21001509 | 16 | 5,89382611 |
|  |  | 7 | ASGA0031822 | 21349121 | 16 | 5,54634842 |
|  |  | 7 | ALGA0039404 | 21366193 | 16 | **11,5256118** |
|  |  | 7 | ALGA0039405 | 21385114 | 16 | **11,5256118** |
|  |  | 7 | ASGA0031847 | 21673794 | 16 | **8,48934777** |
|  |  | 7 | ALGA0039447 | 21940478 | 16 | **11,5256118** |
|  |  | 7 | ALGA0039452 | 22008244 | 16 | **8,48934777** |
|  |  | 7 | ALGA0039480 | 22184943 | 16 | 5,15608547 |
|  |  | 7 | MARC0059955 | 22279830 | 16 | 5,32056211 |
|  |  | 7 | ALGA0039611 | 23037876 | 16 | **6,53530185** |
|  |  | 7 | MARC0091468 | 23110906 | 16 | **7,38278781** |
|  |  | 7 | DIAS0001001 | 23153002 | 16 | 4,4984136 |
|  |  | 7 | ALGA0039615 | 23177023 | 16 | **8,56953954** |
|  |  | 7 | MARC0089437 | 24217931 | 16 | **10,3264934** |
|  |  | 7 | M1GA0009803 | 24259597 | 16 | **6,67827318** |
|  |  | 7 | MARC0024470 | 24296032 | 16 | **6,88076391** |
|  |  | 7 | MARC0058875 | 24865366 | 16 | 4,57481642 |
|  |  | 7 | ASGA0032099 | 24944510 | 16 | **6,71286365** |
|  |  | 7 | H3GA0020505 | 25049757 | 16 | 5,5746533 |
|  |  | 7 | ASGA0032123 | 25400951 | 16 | **6,06412963** |
|  |  | 7 | ASGA0032135 | 25489746 | 16 | **6,68154431** |
|  |  | 7 | ALGA0039804 | 25516781 | 16 | 5,6682509 |
|  |  | 7 | DRGA0007396 | 25565917 | 16 | **12,5754271** |
|  |  | 7 | ALGA0039819 | 25591627 | 16 | **6,68154431** |
|  |  | 7 | ALGA0039826 | 25692312 | 16 | **6,68154431** |
|  |  | 7 | ALGA0039828 | 25698413 | 16 | **6,68154431** |
|  |  | 7 | MARC0087333 | 25830498 | 16 | **11,0074991** |
|  |  | 7 | ALGA0039842 | 25872510 | 16 | **11,0074991** |
|  |  | 7 | ASGA0032151 | 25967157 | 16 | **5,96406221** |
|  |  | 7 | DRGA0007412 | 26002978 | 16 | 4,4534376 |
|  |  | 7 | DRGA0007413 | 26015939 | 16 | **10,9271364** |
|  |  | 7 | INRA0024524 | 26069284 | 16 | **12,4513195** |
|  |  | 7 | DRGA0007416 | 26149917 | 16 | **6,60477848** |
|  |  | 7 | ASGA0032174 | 26433052 | 16 | **6,1667056** |
|  |  | 7 | ALGA0039866 | 26451150 | 16 | **6,77569489** |
|  |  | 7 | BGIS0008247 | 26522116 | 16 | 5,81083827 |
|  |  | 7 | ASGA0032239 | 27371495 | 16 | **6,12425483** |
|  |  | 7 | ALGA0040000 | 27707775 | 16 | **6,11647597** |
|  |  | 7 | ASGA0032255 | 27765368 | 16 | **6,12425483** |
|  |  | 7 | ASGA0032257 | 27905935 | 16 | **6,12425483** |
|  |  | 7 | M1GA0009851 | 28029699 | 16 | **10,0211578** |
|  |  | 7 | DRGA0007462 | 28072350 | 16 | **6,12425483** |
|  |  | 7 | ASGA0032266 | 28115845 | 16 | **6,69405135** |
|  |  | 7 | M1GA0009853 | 28212506 | 16 | **6,53863055** |
|  |  | 7 | ASGA0032282 | 28252780 | 16 | **10,4411391** |
|  |  | 7 | H3GA0020623 | 28383120 | 16 | **6,86687323** |
|  |  | 7 | MARC0063300 | 28451792 | 16 | **10,4411391** |
|  |  | 7 | H3GA0020641 | 28521421 | 16 | **6,69405135** |
|  |  | 7 | ASGA0032304 | 28541774 | 16 | **6,69405135** |
|  |  | 7 | ALGA0040052 | 28559177 | 16 | 5,27391126 |
|  |  | 7 | ASGA0032313 | 28625619 | 16 | **6,69405135** |
|  |  | 7 | ASGA0032316 | 28655399 | 16 | 5,50162327 |
|  |  | 7 | ALGA0040066 | 28721664 | 16 | 5,50162327 |
|  |  | 7 | ASGA0032320 | 28744265 | 16 | **10,4411391** |
|  |  | 7 | ASGA0032322 | 28798510 | 16 | **6,86687323** |
|  |  | 7 | ALGA0040076 | 28837944 | 16 | **6,69405135** |
|  |  | 7 | M1GA0009865 | 28897345 | 16 | **10,4411391** |
|  |  | 7 | ALGA0040085 | 28954803 | 16 | **6,69405135** |
|  |  | 7 | ALGA0119762 | 28998392 | 16 | **6,69405135** |
|  |  | 7 | ALGA0109063 | 29005790 | 16 | **6,69405135** |
|  |  | 7 | MARC0012864 | 29006040 | 16 | **5,91464519** |
|  |  | 7 | ASGA0032336 | 29060950 | 16 | 5,27391126 |
|  |  | 7 | ASGA0032342 | 29107207 | 16 | **10,8915128** |
|  |  | 7 | ALGA0040094 | 29122742 | 16 | **6,86687323** |
|  |  | 7 | MARC0068518 | 29166478 | 16 | **10,8915128** |
|  |  | 7 | ALGA0040113 | 29197718 | 16 | **10,8915128** |
|  |  | 7 | SIRI0000873 | 29228734 | 16 | **6,69405135** |
|  |  | 7 | M1GA0009879 | 29268803 | 16 | **10,8915128** |
|  |  | 7 | ALGA0040120 | 29289201 | 16 | **6,61969829** |
|  |  | 7 | MARC0079017 | 29372986 | 16 | **10,8778464** |
|  |  | 7 | ASGA0032398 | 29422512 | 16 | **6,56607983** |
|  |  | 7 | ALGA0040139 | 29442694 | 16 | **6,56607983** |
|  |  | 7 | ALGA0040140 | 29462756 | 16 | 4,71903713 |
|  |  | 7 | ALGA0040148 | 29487703 | 16 | **6,56607983** |
|  |  | 7 | ALGA0040153 | 29522461 | 16 | **6,56607983** |
|  |  | 7 | H3GA0020718 | 29707157 | 16 | **6,49883115** |
|  |  | 7 | M1GA0009910 | 29744642 | 16 | **6,6443761** |
|  |  | 7 | SIRI0000046 | 29878705 | 16 | 4,71903713 |
|  |  | 7 | ALGA0040227 | 30176520 | 16 | **6,65980384** |
|  |  | 7 | MARC0033464 | 30572315 | 16 | **6,06752065** |
|  |  | 7 | ALGA0040298 | 30893735 | 16 | 4,34022922 |
|  |  | 7 | H3GA0020852 | 31400744 | 16 | **6,62251162** |
|  |  | 7 | DIAS0004649 | 32209256 | 16 | **7,64838726** |
|  |  | 7 | ALGA0040423 | 32827483 | 16 | **11,218315** |
|  |  | 7 | MARC0014933 | 33216744 | 16 | **8,07413533** |
|  |  | 7 | INRA0024930 | 34123154 | 16 | **7,3524586** |
|  |  | 7 | ALGA0040524 | 34151758 | 16 | **7,3524586** |
|  |  | 7 | DIAS0000554 | 34166932 | 16 | **14,0441262** |
|  |  | 7 | ASGA0032785 | 34422959 | 16 | **7,34003154** |
|  |  | 7 | H3GA0020988 | 34502131 | 16 | **7,34195138** |
|  |  | 7 | ALGA0040598 | 34772625 | 16 | **6,12516984** |
|  |  | 7 | MARC0073134 | 34786446 | 16 | **13,144097** |
|  |  | 7 | ASGA0032847 | 34835490 | 16 | 5,61823892 |
|  |  | 7 | H3GA0021033 | 34899140 | 16 | 4,31988396 |
|  |  | 7 | MARC0051108 | 35135602 | 16 | 4,99037916 |
|  |  | 7 | MARC0044921 | 35135926 | 16 | **12,3247351** |
|  |  | 7 | ALGA0040640 | 35268388 | 16 | 5,43351183 |
|  |  | 7 | INRA0025056 | 35394560 | 16 | 5,18179787 |
|  |  | 7 | ALGA0040661 | 35411077 | 16 | 4,86840058 |
|  |  | 7 | MARC0015804 | 35436482 | 16 | 4,91552382 |
|  |  | 7 | ALGA0040695 | 35641882 | 16 | **6,6670064** |
|  |  | 7 | ASGA0032963 | 35667542 | 16 | **8,1331452** |
|  |  | 7 | ALGA0040717 | 35670068 | 16 | **6,6670064** |
|  |  | 7 | H3GA0021114 | 35747117 | 16 | **6,52691028** |
|  |  | 7 | ALGA0040721 | 35785388 | 16 | **7,25317594** |
|  |  | 7 | ALGA0040739 | 35970410 | 16 | **6,16052635** |
|  |  | 7 | ALGA0040759 | 36161073 | 16 | 5,57185872 |
|  |  | 7 | ALGA0040772 | 36203824 | 16 | 4,63811547 |
|  |  | 7 | ALGA0040777 | 36323988 | 16 | 5,25155923 |
|  |  | 7 | INRA0025107 | 36446715 | 16 | **9,25273505** |
|  |  | 7 | MARC0060135 | 36587451 | 16 | **8,37014003** |
|  |  | 7 | ASGA0033035 | 36614274 | 16 | 4,82989971 |
|  |  | 7 | H3GA0021185 | 36795710 | 16 | **8,68879094** |
|  |  | 7 | H3GA0021205 | 37145252 | - | 4,82989971 |
|  |  | 7 | DBMA0000241 | 39303646 | - | 5,73886565 |
|  |  | 7 | ALGA0040937 | 39316386 | - | 5,73886565 |
|  |  | 7 | ALGA0041064 | 41243726 | - | 4,57933147 |
|  |  | 7 | ASGA0033347 | 41971593 | - | 4,35575546 |
|  |  | 13 | MARC0080097 | 96537378 | - | **6,69405135** |
| CRCL | D3 | 1 | ASGA0007292 | 260256060 | - | 4,9542234 |
|  |  | 1 | ALGA0009914 | 260280496 | - | 4,83375051 |
|  |  | 1 | H3GA0004688 | 261548558 | - | 5,34457245 |
|  |  | 1 | H3GA0004844 | 264256520 | - | 4,52484475 |
|  |  | 1 | H3GA0004851 | 264309057 | - | 4,88278838 |
|  |  | 1 | INRA0007591 | 264894199 | - | 5,32036954 |
|  |  | 1 | ASGA0007588 | 265031489 | - | 4,55159516 |
|  |  | 1 | H3GA0004878 | 265179997 | - | 5,65362746 |
|  |  | 1 | ASGA0104483 | 266730368 | - | 5,05389226 |
|  |  | 1 | ALGA0010487 | 267132656 | - | 4,37806385 |
|  |  | 1 | H3GA0005226 | 270771716 | - | 5,33430553 |
| CRCL | D1D2D3 | 1 | INRA0007591 | 264894199 | - | 5,02514902 |
|  |  | 7 | DIAS0002244 | 21001509 | - | 5,06464794 |
|  |  | 7 | DIAS0004622 | 23659424 | 17 | **6,7032734** |
|  |  | 7 | DIAS0002191 | 23970744 | 17 | 5,65631181 |
|  |  | 7 | H3GA0020462 | 24141402 | 17 | 4,44304414 |
|  |  | 7 | M1GA0009803 | 24259597 | 17 | **6,07564627** |
|  |  | 7 | MARC0024470 | 24296032 | 17 | **7,08541569** |
|  |  | 7 | ASGA0032099 | 24944510 | 17 | **7,53492811** |
|  |  | 7 | H3GA0020505 | 25049757 | 17 | 4,32360456 |
|  |  | 7 | BGIS0008247 | 26522116 | 17 | **7,15176715** |
|  |  | 7 | ASGA0032316 | 28655399 | - | 4,42064062 |
|  |  | 7 | ASGA0032336 | 29060950 | - | 4,41502686 |
|  |  | 7 | ALGA0040238 | 30197014 | - | 4,93390899 |
|  |  | 7 | DIAS0004649 | 32209256 | - | **6,1078486**1 |
|  |  | 7 | MARC0014933 | 33216744 | - | 4,76103858 |
|  |  | 7 | INRA0024902 | 33639993 | - | 4,57685575 |
|  |  | 7 | ALGA0040524 | 34151758 | - | 5,20316212 |
|  |  | 7 | ASGA0032785 | 34422959 | - | 4,51486655 |
|  |  | 7 | MARC0015804 | 35436482 | - | 4,94867379 |
|  |  | 7 | H3GA0022644 | 97147161 | 18 | **6,53716643** |
|  |  | 7 | DRGA0008026 | 97195350 | 18 | **9,16159928** |
|  |  | 7 | ALGA0043941 | 97247184 | 18 | **11,0762929** |
|  |  | 7 | H3GA0022648 | 97279129 | 18 | **6,13561234** |
|  |  | 7 | INRA0027623 | 97521999 | 18 | **8,41576266** |
|  |  | 7 | ALGA0043984 | 99116565 | 18 | 4,52249529 |
|  |  | 7 | ALGA0044022 | 99337831 | 18 | 4,60581633 |
|  |  | 7 | ASGA0035589 | 99414005 | 18 | **6,36234119** |
|  |  | 7 | ASGA0035590 | 99424987 | 18 | **6,40011475** |
|  |  | 7 | H3GA0022712 | 99491117 | - | 4,80913067 |
|  |  | 7 | ASGA0035609 | 99677153 | - | 5,10568837 |
|  |  | 7 | H3GA0022720 | 99705755 | - | 5,41912518 |
|  |  | 7 | ALGA0044121 | 100545566 | - | 5,04692925 |
|  |  | 7 | ALGA0044233 | 102302976 | - | 5,43698431 |
|  |  | 17 | H3GA0047937 | 13692477 | 19 | **6,45836834** |
|  |  | 17 | ASGA0089601 | 13979012 | 19 | **6,3641113** |
|  |  | 17 | ALGA0103055 | 14252000 | 19 | **6,21803291** |
|  |  | 17 | MARC0056509 | 15256964 | 19 | **10,7026116** |
|  |  | 17 | ALGA0093434 | 15321777 | 19 | **6,6620917** |
|  |  | 17 | ASGA0075539 | 15389339 | 19 | **6,3617654** |
|  |  | 17 | ALGA0093437 | 15401211 | 19 | **6,86311145** |
|  |  | 17 | ALGA0093440 | 15421803 | 19 | **5,92408459** |
|  |  | 17 | INRA0052780 | 15659761 | 19 | 4,33274883 |
|  |  | 17 | MARC0074172 | 15768175 | 19 | **11,1226269** |
|  |  | 17 | INRA0052808 | 15896846 | 19 | **15,7715817** |
|  |  | 17 | ASGA0075556 | 16422085 | 19 | **9,09090793** |
|  |  | 17 | MARC0028591 | 16634318 | 19 | 4,49946577 |
|  |  | 17 | ALGA0105626 | 16828543 | 19 | **5,95123978** |
|  |  | 17 | ALGA0093478 | 16919581 | 19 | **17,8849989** |
|  |  | 17 | ALGA0093481 | 16940859 | 19 | **8,56564989** |
|  |  | 17 | ALGA0093482 | 16966248 | 19 | 4,75396205 |
|  |  | 17 | ALGA0093570 | 18039052 | 19 | 5,0646424 |
|  |  | 17 | ALGA0093616 | 18543305 | 19 | **7,83858408** |
|  |  | 17 | ASGA0075671 | 18738724 | 19 | 4,52756098 |
|  |  | 17 | H3GA0048042 | 19474175 | 19 | **6,64070345** |
|  |  | 17 | DRGA0016627 | 20677863 | - | 4,42219121 |
|  |  | 17 | ASGA0075733 | 21298973 | - | 5,45915893 |
| CRCL | MA | 1 | INRA0007591 | 264894199 | - | 5,51018209 |
|  |  | 7 | DIAS0004622 | 23659424 | - | 5,16165473 |
|  |  | 7 | DIAS0002191 | 23970744 | - | 4,38268507 |
|  |  | 7 | H3GA0022644 | 97147161 | 20 | **6,88239731** |
|  |  | 7 | DRGA0008026 | 97195350 | 20 | **7,35625131** |
|  |  | 7 | ALGA0043941 | 97247184 | 20 | **10,1003436** |
|  |  | 7 | H3GA0022648 | 97279129 | 20 | 4,96018945 |
|  |  | 7 | INRA0027623 | 97521999 | 20 | **7,42285302** |
|  |  | 7 | ALGA0043984 | 99116565 | 20 | 4,62269375 |
|  |  | 7 | ALGA0044022 | 99337831 | 20 | 5,67551177 |
|  |  | 7 | ASGA0035589 | 99414005 | 20 | **7,70531338** |
|  |  | 7 | ASGA0035590 | 99424987 | 20 | **7,71556927** |
|  |  | 7 | H3GA0022712 | 99491117 | 20 | **6,35193287** |
|  |  | 7 | ASGA0035609 | 99677153 | - | 4,69271795 |
|  |  | 7 | H3GA0022720 | 99705755 | - | 5,69853593 |
|  |  | 7 | H3GA0022724 | 99887568 | - | 4,67881597 |
|  |  | 7 | ALGA0044121 | 100545566 | - | 5,09291125 |
|  |  | 7 | ALGA0044233 | 102302976 | - | 5,35951856 |
|  |  | 17 | ALGA0092525 | 1066109 | - | 4,80631897 |
|  |  | 17 | MARC0061426 | 1135064 | - | 4,43794517 |
|  |  | 17 | ALGA0092584 | 1734092 | - | 4,635449 |
|  |  | 17 | ASGA0074980 | 1971412 | - | 5,12073204 |
|  |  | 17 | DRGA0016546 | 9953781 | - | 4,53402263 |
|  |  | 17 | ALGA0093177 | 10072333 | - | 4,30129065 |
|  |  | 17 | ASGA0100556 | 10177122 | - | 4,40197593 |
|  |  | 17 | ALGA0093121 | 11511966 | - | 4,64801054 |
|  |  | 17 | H3GA0047937 | 13692477 | 19 | **6,47586362** |
|  |  | 17 | ASGA0089601 | 13979012 | 19 | **6,50487212** |
|  |  | 17 | ALGA0103055 | 14252000 | 19 | 5,72653573 |
|  |  | 17 | ASGA0075536 | 15196027 | 19 | **6,71287038** |
|  |  | 17 | MARC0056509 | 15256964 | 19 | **11,8068754** |
|  |  | 17 | ALGA0093434 | 15321777 | 19 | **9,06706986** |
|  |  | 17 | ASGA0075539 | 15389339 | 19 | **6,34476549** |
|  |  | 17 | ALGA0093437 | 15401211 | 19 | **9,50709999** |
|  |  | 17 | ALGA0093440 | 15421803 | 19 | **7,84224111** |
|  |  | 17 | INRA0052780 | 15659761 | 19 | 5,00524306 |
|  |  | 17 | MARC0112426 | 15755718 | 19 | 5,03058409 |
|  |  | 17 | MARC0074172 | 15768175 | 19 | **11,5086383** |
|  |  | 17 | INRA0052808 | 15896846 | 19 | **17,7079656** |
|  |  | 17 | CASI0008233 | 15904945 | 19 | 5,39718066 |
|  |  | 17 | ALGA0093449 | 15919300 | 19 | 5,33264045 |
|  |  | 17 | ALGA0093461 | 16279385 | 19 | 4,33941909 |
|  |  | 17 | ASGA0075556 | 16422085 | 19 | **10,1919241** |
|  |  | 17 | MARC0028591 | 16634318 | 19 | 5,73329803 |
|  |  | 17 | ALGA0105626 | 16828543 | 19 | **7,37314659** |
|  |  | 17 | ALGA0093478 | 16919581 | 19 | **19,0826521** |
|  |  | 17 | ALGA0093481 | 16940859 | 19 | **9,58854866** |
|  |  | 17 | ALGA0093482 | 16966248 | 19 | **7,20321759** |
|  |  | 17 | ALGA0093484 | 17075321 | 19 | 4,73329803 |
|  |  | 17 | ALGA0093570 | 18039052 | 19 | 4,84863015 |
|  |  | 17 | ALGA0093616 | 18543305 | 19 | **9,34814073** |
|  |  | 17 | ASGA0075671 | 18738724 | 19 | 4,77676373 |
|  |  | 17 | H3GA0048042 | 19474175 | 19 | **6,89008414** |
|  |  | 17 | ASGA0097904 | 19639240 | - | 5,11633856 |
|  |  | 17 | ASGA0075694 | 19679617 | - | 4,53865157 |
|  |  | 17 | MARC0058896 | 23773788 | - | 5,17528856 |
|  |  | 17 | MARC0061206 | 28823286 | - | 5,05482695 |
