# Supplementary material for: Genome-wide association studies and meta-analysis uncovers new candidate genes for growth and carcass traits in pigs
Source: PLoS One. 2018 Oct 11;13(10):e0205576. doi: 10.1371/journal.pone.0205576 (PMC6181390; doi:10.1371/journal.pone.0205576)
Supplement: S2 Table — All significant at p < 0.05, except for the ones marked as ns = not significant. (DOCX) [file pone.0205576.s003.docx]

**S2 Table.** **Pearson's product-moment correlation for *p* values of the common markers in D1, D2, D3, D1D2D3 GWAS output and MA output.**

All significant at $p<0.05$, except for the ones marked as ns = not significant.

| **ADG** | D1 | D2 | D3 | D1D2D3 | MA |
| --- | --- | --- | --- | --- | --- |
| D1 |  | 0.02 | ns | 0.45 | 0.64 |
| D2 |  |  | ns | 0.19 | 0.10 |
| D3 |  |  |  | 0.08 | 0.08 |
| D1D2D3 |  |  |  |  | 0.83 |
| MA |  |  |  |  |  |
| **BFT** | D1 | D2 | D3 | D1D2D3 | MA |
| D1 |  | 0.04 | ns | 0.53 | 0.58 |
| D2 |  |  | ns | 0.17 | 0.10 |
| D3 |  |  |  | 0.08 | 0.17 |
| D1D2D3 |  |  |  |  | 0.81 |
| MA |  |  |  |  |  |
| **MFR** | D1 | D2 | D3 | D1D2D3 | MA |
| D1 |  | 0.04 | 0.03 | 0.39 | 0.68 |
| D2 |  |  | ns | 0.23 | 0.09 |
| D3 |  |  |  | 0.13 | 0.14 |
| D123 |  |  |  |  | 0.74 |
| MA |  |  |  |  |  |
| **CRCL** | D1 | D2 | D3 | D1D2D3 | MA |
| D1 |  | ns | -0.03 | 0.50 | 0.55 |
| D2 |  |  | 0.01 | 0.18 | 0.07 |
| D3 |  |  |  | 0.05 | 0.16 |
| D1D2D3 |  |  |  |  | 0.74 |
| MA |  |  |  |  |  |
